# Estimates and Projections of the Global Economic Cost of 29 Cancers in 204 Countries and Territories From 2020 to 2050

**DOI:** 10.1001/jamaoncol.2022.7826

**Published:** 2023-02-23

**Authors:** Simiao Chen, Zhong Cao, Klaus Prettner, Michael Kuhn, Juntao Yang, Lirui Jiao, Zhuoran Wang, Weimin Li, Pascal Geldsetzer, Till Bärnighausen, David E. Bloom, Chen Wang

**Affiliations:** 1 Heidelberg Institute of Global Health, Faculty of Medicine and University Hospital, Heidelberg University, Heidelberg, Germany; 2 Chinese Academy of Medical Sciences and Peking Union Medical College, Beijing, China; 3 Institute for Artificial Intelligence, Tsinghua University, Beijing, China; 4 State Key Lab of Intelligent Technologies and Systems, Beijing National Research Center for Information Science and Technology, Department of Automation, Tsinghua University, Beijing, China; 5 Vienna Institute of Demography, Wittgenstein Centre, International Institute for Applied Systems Analysis, OeAW, University of Vienna, Vienna, Austria; 6 Vienna University of Economics and Business (WU), Department of Economics, Vienna, Austria; 7 International Institute for Applied Systems Analysis, Laxenburg, Austria; 8 State Key Laboratory of Medical Molecular Biology, Institute of Basic Medical Sciences, Chinese Academy of Medical Sciences and Peking Union Medical College, Beijing, China; 9 Columbia Mailman School of Public Health, New York, New York; 10 Department of Respiratory and Critical Care Medicine, West China Hospital, Sichuan University, Sichuan, China; 11 Institute of Respiratory Health, Frontiers Science Center for Disease-related Molecular Network, West China Hospital, Sichuan University, Sichuan, China; 12 Division of Primary Care and Population Health, Department of Medicine, Stanford University, Stanford, California; 13 Department of Global Health and Population, Harvard T. H. Chan School of Public Health, Boston, Massachusetts; 14 National Clinical Research Center for Respiratory Diseases, Beijing, China; 15 Chinese Academy of Engineering, Beijing, China

## Abstract

**Importance:**

Cancers are a leading cause of mortality, accounting for nearly 10 million annual deaths worldwide, or 1 in 6 deaths. Cancers also negatively affect countries’ economic growth. However, the global economic cost of cancers and its worldwide distribution have yet to be studied.

**Objective:**

To estimate and project the economic cost of 29 cancers in 204 countries and territories.

**Design, Setting, and Participants:**

A decision analytical model that incorporates economic feedback in assessing health outcomes associated with the labor force and investment. A macroeconomic model was used to account for (1) the association of cancer-related mortality and morbidity with labor supply; (2) age-sex-specific differences in education, experience, and labor market participation of those who are affected by cancers; and (3) the diversion of cancer treatment expenses from savings and investments. Data were collected on April 25, 2022.

**Main Outcomes and Measures:**

Economic cost of 29 cancers across countries and territories. Costs are presented in international dollars at constant 2017 prices.

**Results:**

The estimated global economic cost of cancers from 2020 to 2050 is $25.2 trillion in international dollars (at constant 2017 prices), equivalent to an annual tax of 0.55% on global gross domestic product. The 5 cancers with the highest economic costs are tracheal, bronchus, and lung cancer (15.4%); colon and rectum cancer (10.9%); breast cancer (7.7%); liver cancer (6.5%); and leukemia (6.3%). China and the US face the largest economic costs of cancers in absolute terms, accounting for 24.1% and 20.8% of the total global burden, respectively. Although 75.1% of cancer deaths occur in low- and middle-income countries, their share of the economic cost of cancers is lower at 49.5%. The relative contribution of treatment costs to the total economic cost of cancers is greater in high-income countries than in low-income countries.

**Conclusions and Relevance:**

In this decision analytical modeling study, the macroeconomic cost of cancers was found to be substantial and distributed heterogeneously across cancer types, countries, and world regions. The findings suggest that global efforts to curb the ongoing burden of cancers are warranted.

## Key Points

**Question:**

What is the estimated economic cost and cost distribution of 29 cancers in 204 countries and territories from 2020 to 2050?

**Findings:**

In this decision analytical modeling study, the global economic cost of cancers from 2020 to 2050 was estimated to be $25.2 trillion (in international dollars at constant 2017 prices). The economic burden and the health burden were distributed unequally across countries, world regions, and country income groups.

**Meaning:**

Results of this study suggest that global efforts to contain projected increases in the burden of cancers are warranted.

## Teaser

This decision analytical model study estimates the macroeconomic cost of 29 cancers in 204 countries and territories.

## Introduction

Cancers are a leading cause of death worldwide,^1^ causing 10.0 million deaths in 2019.^2^ The incidence of cancers and resultant mortality are increasing all over the world; this growth reflects population aging^3^ and the role of several risk factors such as tobacco use, alcohol use, unhealthy diet, physical inactivity, and air pollution.^4^ The health burden of cancers is distributed unevenly across countries, with high-income countries facing a greater per-population burden in terms of disability-adjusted life-years (DALYs) than low- and middle-income countries (LMICs).^3^ In 2019, China experienced the largest absolute death toll due to cancers, followed by India and the US. eFigures 1, 2, 3, and 4 in eAppendix 1 in Supplement 1 show the incidence, prevalence, mortality, and years-of-life-lost rates of cancers in detail.

Under the Obama Administration in 2016, then-Vice President Biden launched the Cancer Moonshot with a mission to accelerate progress against cancers.^5^ On February 2, 2022, President Biden announced the reignition of the Cancer Moonshot and highlighted new goals, including a 50% reduction in the death rate from cancers over the next 25 years.^6^ The plan, however, does not yet include any new funding commitments. Other important recent initiatives include the 4th edition of the European Code Against Cancer, which suggests 12 evidence-based approaches to prevent cancers across Europe.^7^ Notable global targets for cancer reduction have also been announced. The World Health Organization (WHO) Global Action Plan for the Prevention and Control of Noncommunicable Diseases for 2013 to 2020 proposed a target of 25% relative reduction in premature mortality from cardiovascular diseases, cancer, diabetes, and chronic respiratory diseases by 2025.^8^ In addition, the United Nations Sustainable Development Goal target 3.4 is to reduce one-third of premature mortality from noncommunicable diseases, including cancer, by 2030, compared with 2015, and to promote mental health and well-being.^9^

Cancers impose a marked toll on the economy through reduced productivity, unemployment, labor losses, and capital investment reductions. Accordingly, investment in cancer screening, diagnosis, and treatment could yield substantial health and economic benefits, especially in LMICs, which have lower levels of cancer survival compared with high-income countries.^10^ Knowing the economic cost of cancers enables policy makers to (1) enact appropriate policies to curb the increase in cancer-related mortality and morbidity, (2) allocate resources appropriately, and (3) build health care systems that can cope effectively with expected increases in cancer prevalence.

Several previous studies^11,12,13^ have assessed the economic cost of cancers for a single or a limited set of countries or cancer types. Two studies^14,15^ estimated and projected the macroeconomic cost of all cancers; however, these studies are 10 or more years old, and the estimates should therefore be updated. Furthermore, most previous approaches have not accounted for the dynamics of morbidity and mortality–related changes in the population and the implications of treatment costs for savings (and thus capital accumulation).^12,16,17,18^

This study estimates the macroeconomic cost of all cancers for most countries in the world, based on a decision analytical model that accounts for productivity losses due to cancer-related mortality and morbidity among people with different educational and experience levels and for changes in savings and investment patterns due to the costs of cancer treatments.

## Methods

### Model Description

This study used publicly accessible data that were not collected specifically for our analysis, does not meet the regulatory definition of human participant research, and was exempt from institutional review board review on the basis of the Common Rule. This study followed the Consolidated Health Economic Evaluation Reporting Standards (CHEERS) reporting guideline. Previous studies^19,20,21,22,23,24,25^ and eAppendix 2 in Supplement 1 describe the health-augmented macroeconomic model for estimating the cost of cancer in detail. The pathways by which cancers affect the economy in the model include the following. First, each type of cancer reduces effective labor supply due to mortality and morbidity. Mortality resulting from cancer reduces the population and therefore the number of working-age individuals, whereas cancer morbidity reduces productivity and increases absenteeism. Second, households with affected family members divert a certain share of their savings to finance out-of-pocket treatment costs. In addition, insurance-funded coverage of cancer treatment costs translates into higher private health insurance premiums and public health insurance taxes, depending on the mode of health insurance financing. Both channels reduce aggregate savings and investments, which decreases economywide physical capital accumulation.

### Statistical Analysis

To quantify the macroeconomic cost of 29 cancers, we compared aggregate output (gross domestic product [GDP]) across 2 scenarios over the period from 2020 to 2050: (1) the status quo scenario with no interventions to reduce the mortality and morbidity of cancers relative to current and projected rates and (2) a counterfactual scenario assuming the complete elimination of cancers. We then calculated the macroeconomic cost of cancers as the cumulative difference in projected annual GDP between these 2 scenarios.

### Data

The definition of each cancer is from the Global Burden of Disease Study cancer categories.^2^ We considered data for 204 countries and territories and for a set of World Bank regions,^2^ representing 99.7% of the world’s population. The GDP and saving rates are from the World Bank World Development Indicators database^26,27^ and the World Economic Outlook database.^28^ Mortality and morbidity data are from Global Burden of Disease 2019.^2^ Human capital is based on the educational attainment projections of Barro and Lee^29^ and workforce experience according to a mincerian specification.^30^ The estimated parameters for the mincerian specification are taken from Psacharopoulos and Patrinos^31^ for educational level and from Heckman et al^32^ for experience. Physical capital data are from the Penn World Table,^33^ with the value for the output elasticity of physical capital following standard economic estimates.^34^ eTable 1 in eAppendix 3 in Supplement 1 describes the parameter values in more detail. All economic data and estimations were converted to 2017 international dollars (INT $).

Treatment costs for each cancer (eAppendix 3 [eTable 2] in Supplement 1) were obtained from Dieleman et al.^35^ eAppendix 3 in Supplement 1 presents more detailed data sources and model assumptions. Of the 204 countries and territories, 60 countries and territories lack the necessary data (eAppendix 4 [eTable 3] in Supplement 1). We imputed the results based on GDP and DALYs (eAppendix 4 [eTable 4] in Supplement 1). eAppendix 4 in Supplement 1 describes the imputation in detail.

### Sensitivity Analysis

We conducted sensitivity analyses by varying the mortality and morbidity rates based on the lower and upper bounds (95% uncertainty intervals) of Global Burden of Disease mortality and morbidity data. We provide the 2% discounted estimates in the Results section and eAppendix 5 (eTable 5) in Supplement 1 and results discounted by 0% and 3% in eAppendix 5 (eTables 6-9) in Supplement 1. Analyses were conducted using Python 3.9.1 software (Anaconda Inc).

## Results

### Economic Cost of Included Cancers by Country

**Figure 1.**
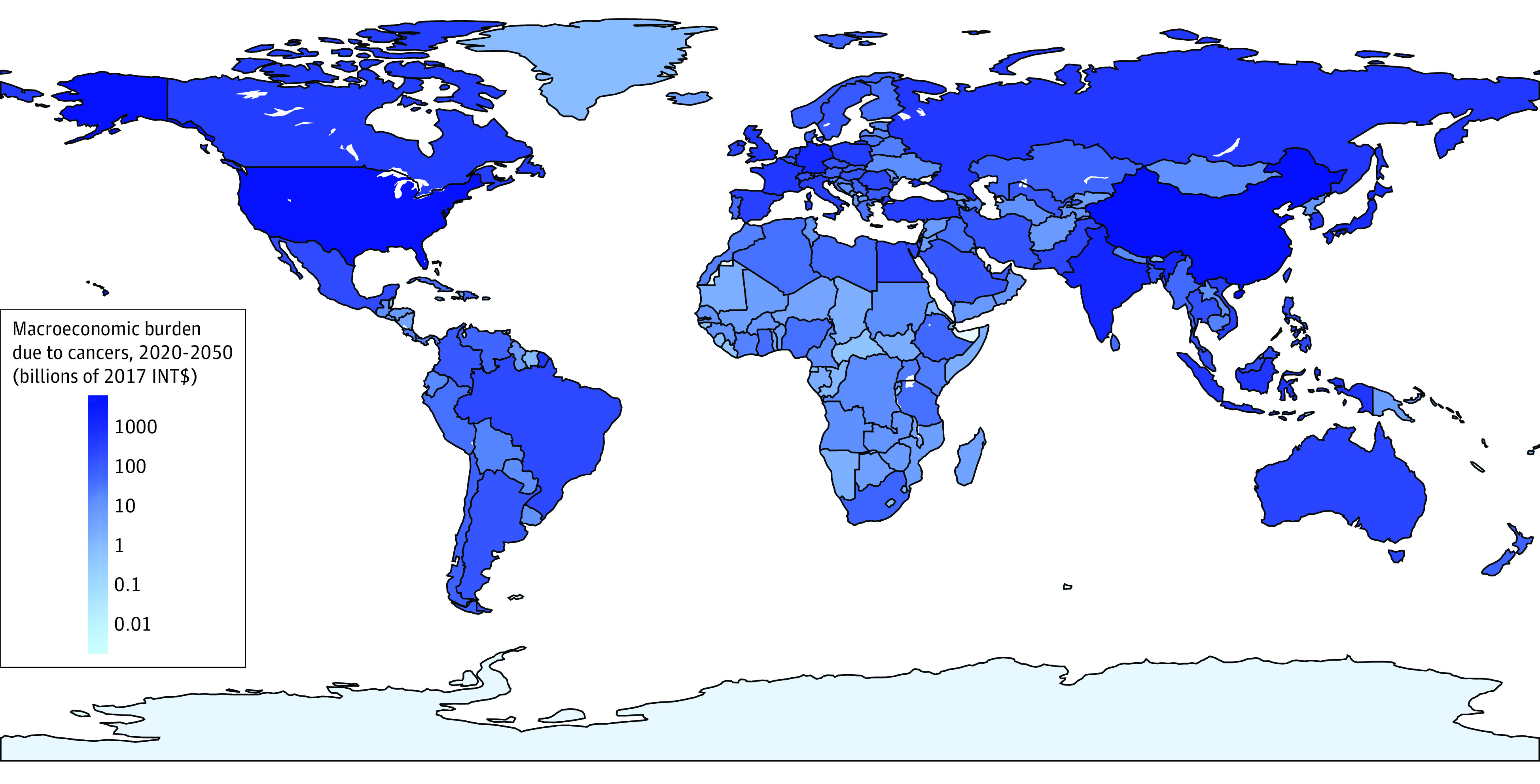
Macroeconomic Burden Due to Cancers From 2020 to 2050 INT$ indicates international dollars at constant 2017 prices.

Figure 1 shows the geographic distribution of the economic burden of cancers in terms of aggregate GDP. eAppendix 5 (eFigure 5) in Supplement 1 presents the results in percentage terms. eAppendix 5 (eTable 5) in Supplement 1 also displays the detailed results for the 144 countries with complete data, which represent 92.7% of the world’s population, and the imputed results for the 60 countries with incomplete data. Of all countries, China faces the largest economic cost of cancers at INT $6.1 trillion, followed by the US (INT $5.3 trillion) and India (INT $1.4 trillion). Bulgaria bears the highest economic cost as a share of GDP at 1.42%, followed by Monaco (1.33%) and Montenegro (1.09%). The per capita economic cost is highest in Monaco at INT $85 230, followed by Ireland (INT $54 009) and Bermuda (INT $20 732). Globally, we estimate the macroeconomic cost of cancers to be INT $25.2 trillion from 2020 to 2050. This result is INT $37.9 trillion if discounted at 0% or INT $20.7 trillion if discounted at 3% (eAppendix 5 [eTables 6 and 7] in Supplement 1). Our results suggest that the economic cost of cancers is equivalent to an annual tax of 0.55% on global output, or a per capita burden of INT $2857 from 2020 to 2050.

### Economic Costs of Individual Cancer Types

Table 1 presents the results for each cancer. Of all cancers, tracheal, bronchus, and lung (TBL) cancer exacts the largest economic cost at INT $3.9 trillion, followed by colon and rectum cancer (INT $2.8 trillion), breast cancer (INT $2.0 trillion), liver cancer (INT $1.7 trillion), and leukemia (INT $1.6 trillion). Across cancer types, TBL cancer (15.4%), colon and rectum cancer (10.9%), breast cancer (7.7%), liver cancer (6.5%), and leukemia (6.3%) account for half of the global economic cost of cancers. The economic cost of TBL cancer is 56 times that of mesothelioma, which causes the lowest economic burden as a percentage of GDP at 0.001% (compared with 0.085% for TBL) and on a per capita basis at INT $7.8 (compared with INT $440.3 for TBL).

**Table 1.** Total Macroeconomic Cost, Macroeconomic Cost as a Share of GDP, and Per Capita Economic Cost in 2020-2050 by Cancer Type

Cancer type	Economic cost in billions of 2017 INT $ (95% UI)	Percentage of total GDP in 2020-2050 (95% UI)	Per capita loss in 2017 INT $ (95% UI)
Tracheal, bronchus, and lung	3888 (2771-5478)	0.085 (0.060-0.120)	440.3 (313.8-620.3)
Colon and rectum	2760 (2012-3749)	0.060 (0.044-0.082)	312.6 (227.8-424.5)
Breast	1964 (1402-2759)	0.043 (0.031-0.060)	222.4 (158.8-312.5)
Liver	1653 (1072-2400)	0.036 (0.023-0.052)	187.2 (121.5-271.8)
Leukemia	1597 (1233-2097)	0.035 (0.027-0.046)	180.8 (139.7-237.5)
Stomach	1449 (1032-2022)	0.032 (0.023-0.044)	164.1 (116.9-229.0)
Brain and central nervous system	1404 (934-1960)	0.031 (0.020-0.043)	159.0 (105.8-221.9)
Pancreatic	1293 (939-1754)	0.028 (0.020-0.038)	146.4 (106.3-198.6)
Other neoplasms	1080 (1029-1155)	0.024 (0.022-0.025)	122.3 (116.5-130.8)
Non-Hodgkin lymphoma	1071 (833-1400)	0.023 (0.018-0.031)	121.3 (94.4-158.5)
Esophageal	827 (618-1197)	0.018 (0.013-0.026)	93.7 (70.0-135.6)
Cervical	682 (406-1034)	0.015 (0.009-0.023)	77.2 (46.0-117.1)
Prostate	624 (473-905)	0.014 (0.010-0.020)	70.7 (53.5-102.5)
Lip and oral cavity	530 (339-820)	0.012 (0.007-0.018)	60.1 (38.4-92.8)
Kidney	536 (377-758)	0.012 (0.008-0.017)	60.7 (42.7-85.8)
Ovarian	519 (350-755)	0.011 (0.008-0.016)	58.7 (39.7-85.5)
Multiple myeloma	404 (319-538)	0.009 (0.007-0.012)	45.8 (36.1-60.9)
Bladder	434 (328-584)	0.009 (0.007-0.013)	49.1 (37.2-66.1)
Non-melanoma skin	378 (340-428)	0.008 (0.007-0.009)	42.8 (38.5-48.4)
Other pharynx	348 (217-540)	0.008 (0.005-0.012)	39.4 (24.6-61.1)
Malignant skin melanoma	337 (216-574)	0.007 (0.005-0.013)	38.1 (24.5-65.0)
Nasopharynx	295 (184-458)	0.006 (0.004-0.010)	33.4 (20.8-51.9)
Larynx	262 (178-391)	0.006 (0.004-0.009)	29.6 (20.2-44.3)
Gallbladder and biliary tract	224 (150-342)	0.005 (0.003-0.007)	25.4 (16.9-38.8)
Uterine	193 (143-269)	0.004 (0.003-0.006)	21.8 (16.2-30.5)
Testicular	133 (83-211)	0.003 (0.002-0.005)	15.0 (9.4-23.9)
Thyroid	129 (87-193)	0.003 (0.002-0.004)	14.6 (9.9-21.9)
Hodgkin lymphoma	143 (95-229)	0.003 (0.002-0.005)	16.2 (10.7-26.0)
Mesothelioma	69 (46-109)	0.001 (0.001-0.002)	7.8 (5.2-12.3)

Abbreviations: GDP, gross domestic product; INT $, international dollars at constant 2017 prices; UI, uncertainty interval.

**Figure 2.**
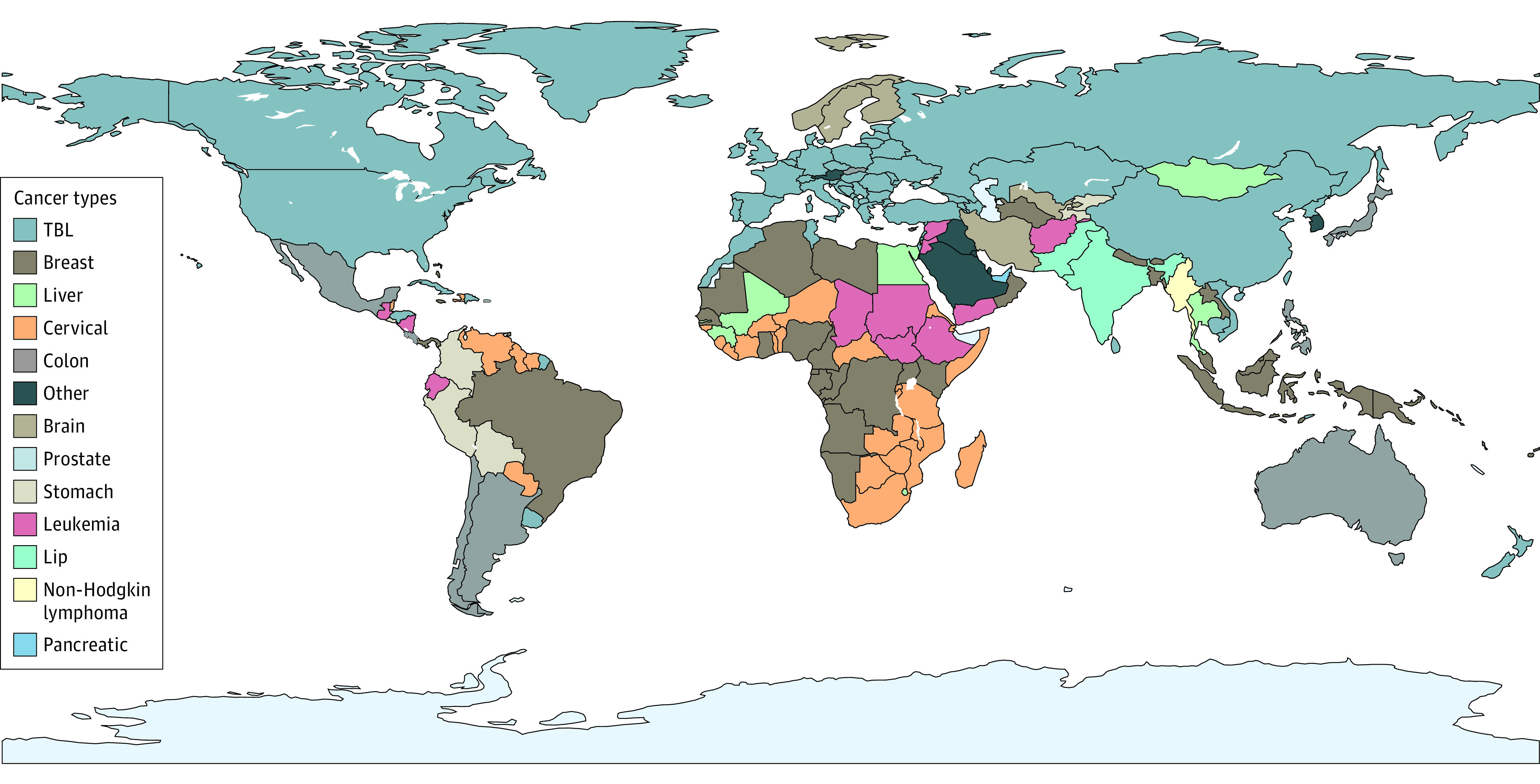
Type of Cancer With the Largest Macroeconomic Cost in Each Country TBL indicates tracheal, bronchus, and lung.

Figure 2 illustrates the type of cancer with the largest macroeconomic cost in each country. The largest burden in most countries, including China and the US, comes from TBL cancer. Slovakia, Japan, and Australia bear costs mainly from colon and rectum cancer. In Brazil and Algeria, breast cancer has the highest macroeconomic cost. India and Pakistan bear economic losses primarily from lip cancer and oral cancer. Stomach cancer imposes the largest economic cost in Colombia, Bolivia, and Peru, whereas liver cancer accounts for the largest cost in Thailand, Egypt, and Mongolia. Within-country rankings of cancer type by economic cost are highly correlated with within-country rankings of cancer type by DALYs (eAppendix 5 [eFigure 6] in Supplement 1) and are affected by other factors such as economic development and regional policies. As income increases, common cancers such as TBL cancer, breast cancer, colon and rectum cancer, and pancreatic cancer become more prevalent than leukemia, cervical cancer, liver cancer, and stomach cancer.

### Distribution of the Economic Cost of Cancers by Region and Income Group

With respect to World Bank regions, Table 2 shows that North America has the highest economic burden from cancers as a percentage of GDP—equivalent to an annual tax of 0.83%—followed by Europe and Central Asia (0.63%) and East Asia and Pacific (0.59%). The lowest burden is 0.24% in Sub-Saharan Africa. Per capita economic cost varies substantially across regions, ranging from INT $241 in Sub-Saharan Africa to INT $14 065 in North America. The aggregate economic cost is highest in East Asia and Pacific at INT $9.7 trillion and second highest in Europe and Central Asia at INT $6.0 trillion. Sub-Saharan Africa faces the lowest burden at INT $395 billion.

**Table 2.** Distribution of the Economic Cost of Cancers by Region and Income Group

Region/income group^a^	Economic cost in billions of 2017 INT $ (95% UI)	Percentage of total GDP in 2020-2050 (95% UI)	Per capita loss in 2017 INT $ (95% UI)
By World Bank region			
East Asia and Pacific	9704 (6595-13 863)	0.593 (0.403-0.848)	3943 (2680-5633)
Europe and Central Asia	5998 (4246-8598)	0.627 (0.444-0.899)	6446 (4562-9240)
Latin America and Caribbean	960 (631-1452)	0.349 (0.229-0.528)	1337 (878-2022)
Middle East and North Africa	727 (445-1178)	0.287 (0.176-0.465)	1294 (791-2096)
North America	5617 (4918-6589)	0.834 (0.730-0.978)	14 065 (12 314-16 498)
South Asia	1821 (1145-2762)	0.294 (0.185-0.446)	863 (543-1308)
Sub-Saharan Africa	395 (226-663)	0.235 (0.135-0.394)	241 (138-404)
By World Bank income group			
High income	12 753 (10 198-16 444)	0.716 (0.572-0.923)	10 294 (8232-13 273)
Upper-middle income	8619 (5686-12 545)	0.535 (0.353-0.779)	3253 (2146-4734)
Lower-middle income	3636 (2207-5739)	0.326 (0.198-0.515)	923 (560-1457)
Low income	168 (92-287)	0.261 (0.143-0.448)	174 (95-298)
Total	25 227 (18 208-35 108)	0.551 (0.397-0.766)	2857 (2062-3976)

Abbreviations: GDP, gross domestic product; INT $, international dollars at constant 2017 prices; UI, uncertainty interval.

^a^ Countries are classified into World Bank regions. as in eTable 5 in Supplement 1. These 7 World Bank regions do not include Cook Islands, Niue, Palestine, and Tokelau.

With respect to income groups, high-income countries bear the greatest burden at 0.72% of total GDP, equivalent to a total economic loss of INT $12.8 trillion and a per capita loss of INT $10 294. By contrast, cancers cost low-income countries 0.26% of GDP, representing INT $168 billion in total and INT $174 in per person losses. eAppendix 5 (eTables 8 and 9) in Supplement 1 presents the discounted estimates by World Bank region and World Bank income group.

We further characterize differences in economic costs across regions and income groups in concert with differences in GDP, population, and DALYs in eAppendix 6 (eTable 10) in Supplement 1. In total, LMICs account for 86.0% of the population, 74.9% of DALYs from cancers in 2020, 61.1% of global GDP, and 49.5% of cancers’ economic cost from 2020 to 2050. As for geographic regions, East Asia and Pacific have the largest population (27.9%), the largest share of DALYs in 2020 (39.1%), and the largest economic cost (38.5%). Europe and Central Asia and North America face the largest per-population DALY burdens in 2050 (column 4 divided by column 6 in eAppendix 6 [eTable 10] in Supplement 1) and the largest economic costs in terms of percentage of total GDP in Table 2.

### Importance of Physical Capital and Human Capital in the Economic Cost of Cancers

We also computed the share of cancers’ total economic burden made up of reductions in physical capital and human capital (eAppendix 7 [eFigures 7- 8] in Supplement 1). Our results suggest that physical capital accumulation plays a more important role in high-income countries than in low-income countries. In high-income countries, reductions in physical capital accumulation and human capital accumulation account for 23.3% and 76.8%, respectively, of the total economic cost due to cancers. These respective shares are 5.0% and 95.1% for low-income countries.

## Discussion

This decision analytical modeling study using the health-augmented macroeconomic model aims to estimate the global economic cost of 29 cancer types for 204 countries and territories. To our knowledge, this study is the first to account for productivity loss among people with different educational and experience levels, unlike the previous World Health Organization EPIC model.^14^ Our findings bridge several knowledge gaps. First, we estimated that between 2020 and 2050, cancers will cost the world economy INT $25.2 trillion, which is equivalent to an annual tax of 0.55% on global GDP during this period and higher than the GDP of China in 2020 (the world’s largest economy in 2017 INT $). Second, our study found that the health and economic costs of cancers were distributed unevenly across cancer types, countries, and regions. Third, our study suggests that investment in cancer mitigation, such as cancer research and development, cost-effectiveness analysis of interventions, and cancer prevention strategies, may yield substantial economic benefits.

Our findings are comparable to results from previous studies on the economic cost of cancers in Europe and some developing countries. A recent study^36^ that used the cost-of-illness approach showed that the total cost of cancers in Europe in 2018 was £199 billion (2017 INT $294 billion). This amount included INT $152 billion in direct costs from cancer-related health expenditures, INT $103 billion in productivity losses, and INT $39 billion in informal care costs.^36^ Our analysis yielded an annual macroeconomic cost of INT $193 billion in Europe and Central Asia from 2020 to 2050 (Table 2). Another study^37^ that applied the incidence-based method estimated that productivity losses due to premature mortality from cancers in Brazil, India, and China amounted to 0.21%, 0.36%, and 0.34% of total GDP in 2012, respectively. In our results, the respective annual macroeconomic burdens from 2020 to 2050 were 0.26%, 0.28%, and 0.62% of GDP (eAppendix 5 [eTable 5 and eFigure 5] in Supplement 1). Productivity loss (defined herein as human capital loss) respectively accounts for 0.24%, 0.26%, and 0.55% of total GDP from 2020 to 2050 in these 3 countries. Indirect costs such as productivity loss due to mortality, morbidity, and informal care are substantial factors in the differences between our results and those of the prior studies. We calculated the direct costs and indirect costs from the accumulation process of human capital and physical capital, and this calculation supports estimating the macroeconomic burden over a longer period than other approaches and the causal relationship between GDP and cancers.

Across regions, East Asia and Pacific face the largest economic toll, followed by Europe and Central Asia and North America. These regions collectively account for 84.5% of the global economic cost of cancers. The high economic burdens these regions face may be due partly to the fact that they are home to the 2 countries with the largest economic cost of cancers: China and the US. The high economic cost of cancers in China is largely attributable to the substantial health burden it faces. China had higher cancer mortality and DALY rates compared with the US.^38,39^ In contrast, in the US, a typical high-income country where people have high educational attainment, high per capita human capital loss is a factor in the large economic cost. Furthermore, the US also faces a high burden in terms of treatment costs and thus substantial loss of physical capital. The spectrum of economic costs from cancers across regions depends on economic factors, environmental characteristics, and social characteristics such as eating habits. For example, high prevalence of betel nut chewing may be a factor in the high economic cost of lip and oral cavity cancer in South Asia.^40^

Across cancer types, TBL cancer (15.4%), colon and rectum cancer (10.9%), breast cancer (7.7%), liver cancer (6.5%), and leukemia (6.3%) account for half of the global economic cost of cancers. Cervical cancer, liver cancer, and leukemia rank first in several LMICs (Figure 2), but not in developed countries. This may be due to developed countries having made greater progress in the prevention, monitoring, and early diagnosis of certain cancers such as those related to infections. Tobacco use is the leading preventable cause of cancer worldwide.^41^ The WHO has promoted policies aimed at reducing tobacco use^42^ and recently reported global progress since 2003 in the implementation of the Framework Convention on Tobacco Control.^43^ However, 49 countries that are home to 2.4 billion people have yet to adopt a single tobacco control policy in the WHO Framework Convention on Tobacco Control.^44^ If China, the leading tobacco producer and consumer, raised taxes on cigarettes to 75% of their retail price and implemented wide-ranging tobacco-control policies, it could save INT $1 trillion from 2015 to 2030.^22^

Differences in economic development may help explain the observed disparities in the magnitude of the economic burden by income group. First, the workforce in high-income countries is typically better educated than that in LMICs, which means that the human capital loss resulting from an equivalent DALY loss will be greater. Second, high-income countries sustain advanced health systems, which suggests much higher treatment costs. Our results suggest that reductions in physical capital accumulation from diverting savings to pay for treatment play a more important role in high-income countries than in low-income countries. Overall, high-income countries bear the highest economic cost of cancers, while LMICs bear the greatest absolute health burden due to their larger populations.

Our findings suggest that investing in effective public health interventions to reduce the burden of cancers is essential for protecting global health and economic well-being. The WHO and the United Nations have issued important guidance for responding to the burden of cancer through increased investment and thoughtful prioritization of relevant policy initiatives,^45,46^ but few countries have made meaningful progress toward international targets for cancer and noncommunicable disease reduction.^47^ Nevertheless, several investment avenues have utility in mitigating the health and economic burdens posed by cancer. First, we need greater investment in medical research and development and promotion of innovation to reduce cancer incidence, prevalence, and mortality. No new funding has yet been made available to relaunch the Cancer Moonshot, and many countries underspend on health generally.^48^ Second, we need more investment in research to identify cost-effective public health interventions. Third, we should strengthen primary care and facilitate the inclusion of cost-effective cancer screening programs in insurance benefit packages. We should also embrace innovative forms of health care delivery, such as community-based screening and digital health. Fourth, we need to strengthen cancer prevention efforts. Effective cancer prevention involves not only the medical system but also other facets of society. For instance, economic measures such as tobacco taxes and laws such as public smoking bans are effective social interventions to reduce tobacco use.

### Limitations

Our model has several limitations. First, we had to rely on imputations or projections to calculate cancer-related health expenditures, labor participation rates, mortality and morbidity, and the missing data for 60 countries. Second, we did not account for changes in the labor force participation of family members of people with cancer requiring informal care, and this may underestimate the economic costs of cancer. Third, we did not include unemployment, and an explicit treatment of price movements and endogenous savings in the framework. eTable 11 in eAppendix 8 in Supplement 1 summarizes the strengths and limitations of the study.

## Conclusions

This study estimated that the global economic cost of cancers from 2020 to 2050 is substantial, equating to a 0.55% tax annually. Of all cancers, TBL exacts the largest economic cost, suggesting an urgency for investment in TBL cancer prevention, diagnosis, control, treatment, and rehabilitation. The economic cost and health burden of cancers are distributed unevenly across cancer types and across countries. High-income countries bear the highest macroeconomic costs. However, LMICs bear the preponderance of the human toll, underscoring the need for improvements on multiple fronts, including health system strengthening and public health policies to reduce tobacco use. Otherwise, as LMICs develop, an economic cost will accompany the human cost they already incur. Our study emphasizes the need to invest in global efforts to contain the cancer burden.

## Notes

Supplement. **eAppendix 1.** Global Health Burden of Cancer **eFigure 1.** All-Cancer Incidence Rate (per 1,000) in 2019 **eFigure 2.** All-Cancer Prevalence Rate (per 1,000) in 2019 **eFigure 3.** All-Cancer Mortality Rate (per 1,000) in 2019 **eFigure 4.** Years of Life Lost to All Cancers (per 1,000) in 2019 **eAppendix 2.** Modeling Details **eAppendix 3.** Data Description **eTable 1.** Parameter Values and Data Sources **eTable 2.** Treatment Costs (Medium, Lower Bound, and Upper Bound) and the Share of all Treatment Cost for all 29 Cancers in the United States **eAppendix 4.** Imputation **eTable 3.** Missing Data for 60 Countries; the Columns Represent Regions, Country Code, Country Name, GDP, Treatment Costs, Education, Health Expenditures, Labor Participation Rate, Physical Capital, Population, and Saving Rate, Respectively **eTable 4.** Estimating the Relationship Among Percentage of Economic Loss, the Two Main PCA Components of IHME Data, and the Indicator of High-Income Countries **eAppendix 5.** Geographic Distribution and Discounted Estimates of Economic Burden of Cancers **eFigure 5.** Economic Cost of Cancers as a Percentage of Total GDP in 2020–2050 **eTable 5.** Total Macroeconomic Cost, Economic Cost as a Share of GDP in 2020–2050, and Per Capita Economic Cost Attributable to Cancers, by World Bank Region and Country (in 2017 INT$), Using Discount Rates of 2% **eTable 6.** Total Macroeconomic Burden Attributable to Cancers in 2020–2050 Using a Discount Rate of 0% for 204 Countries, by World Bank Region (in 2017 INT$) **eTable 7.** Total Macroeconomic Burden Attributable to Cancers in 2020–2050 Using a Discount Rate of 3% for 204 Countries, by World Bank Region (in 2017 INT$) **eFigure 6.** Cancer Type Responsible for the Most DALYs in 2019 for Each Country (TBL = Tracheal, Bronchus, and Lung) **eTable 8.** Total Macroeconomic Cost, Economic Cost as a Share of Total GDP in 2020–2050, and Per Capita Economic Cost Attributable to Cancer Mortality and Morbidity, by World Bank Region, by World Bank Income Group, and Globally With Discount Rate of 0% **eTable 9.** Total Macroeconomic Cost, Economic Cost as a Share of Total GDP in 2020–2050, and Per Capita Economic Cost Attributable to Cancer Mortality and Morbidity, by World Bank Region, by World Bank Income Group, and Globally With Discount Rate of 3% **eAppendix 6.** Differences in Macroeconomic Loss and Lifetime Disease Burden (**eTable 10**) **eTable 10.** Comparison of Macroeconomic Loss and Lifetime Disease Burden by World Bank Region and Country Income Group **eAppendix 7.** Contribution of Treatment Costs and Human Capital **eFigure 7.** Contribution of Treatment Costs to the Total Economic Cost of Cancers by Country Income Group and World Bank Region **eFigure 8.** Contribution of Human Capital to the Total Economic Cost of Cancers by Country Income Group and World Bank Region **eAppendix 8.** Strengths and Limitations **eTable 11.** Strengths and Limitations of Our Model
eReferences

Supplement 2.
Data Sharing Statement
