# Supplementary material for: Estimates and Projections of the Global Economic Cost of 29 Cancers in 204 Countries and Territories From 2020 to 2050
Source: JAMA Oncol. 2023 Feb 23;9(4):465–72. doi: 10.1001/jamaoncol.2022.7826 (PMC9951101; doi:10.1001/jamaoncol.2022.7826)
Supplement: Supplement. — eAppendix 1. Global Health Burden of Cancer eFigure 1. All-Cancer Incidence Rate (per 1,000) in 2019 eFigure 2. All-Cancer Prevalence Rate (per 1,000) in 2019 eFigure 3. All-Cancer Mortality Rate (per 1,000) in 2019 eFigure 4. Years of Life Lost to All Cancers (per 1,000) in 2019 eAppendix 2. Modeling Details eAppendix 3. Data Description eTable 1. Parameter Values and Data Sources eTable 2. Treatment Costs (Medium, Lower Bound, and Upper Bound) and the Share of all Treatment Cost for all 29 Cancers in the United States eAppendix 4. Imputation eTable 3. Missing Data for 60 Countries; the Columns Represent Regions, Country Code, Country Name, GDP, Treatment Costs, Education, Health Expenditures, Labor Participation Rate, Physical Capital, Population, and Saving Rate, Respectively eTable 4. Estimating the Relationship Among Percentage of Economic Loss, the Two Main PCA Components of IHME Data, and the Indicator of High-Income Countries eAppendix 5. Geographic Distribution and Discounted Estimates of Economic Burden of Cancers eFigure 5. Economic Cost of Cancers as a Percentage of Total GDP in 2020–2050 eTable 5. Total Macroeconomic Cost, Economic Cost as a Share of GDP in 2020–2050, and Per Capita Economic Cost Attributable to Cancers, by World Bank Region and Country (in 2017 INT$), Using Discount Rates of 2% eTable 6. Total Macroeconomic Burden Attributable to Cancers in 2020–2050 Using a Discount Rate of 0% for 204 Countries, by World Bank Region (in 2017 INT$) eTable 7. Total Macroeconomic Burden Attributable to Cancers in 2020–2050 Using a Discount Rate of 3% for 204 Countries, by World Bank Region (in 2017 INT$) eFigure 6. Cancer Type Responsible for the Most DALYs in 2019 for Each Country (TBL = Tracheal, Bronchus, and Lung) eTable 8. Total Macroeconomic Cost, Economic Cost as a Share of Total GDP in 2020–2050, and Per Capita Economic Cost Attributable to Cancer Mortality and Morbidity, by World Bank Region, by World Bank Income Group, and Globally With Discoun [file jamaoncol-e227826-s001.pdf]

# Supplemental Online Content

Chen S, Cao Z, Prettner K, et al. Estimates and projections of the global economic cost of 29 cancers in 204 countries and territories from 2020 to 2050. *JAMA Oncol.* Published online February 23, 2023. doi:10.1001/jamaoncol.2022.7826

**eAppendix 1.** Global Health Burden of Cancer

**eFigure 1.** All-Cancer Incidence Rate (per 1,000) in 2019

**eFigure 2.** All-Cancer Prevalence Rate (per 1,000) in 2019

**eFigure 3.** All-Cancer Mortality Rate (per 1,000) in 2019

**eFigure 4.** Years of Life Lost to All Cancers (per 1,000) in 2019

**eAppendix 2.** Modeling Details

**eAppendix 3.** Data Description

**eTable 1.** Parameter Values and Data Sources

**eTable 2.** Treatment Costs (Medium, Lower Bound, and Upper Bound) and the Share of all Treatment Cost for all 29 Cancers in the United States

**eAppendix 4.** Imputation

**eTable 3.** Missing Data for 60 Countries; the Columns Represent Regions, Country Code, Country Name, GDP, Treatment Costs, Education, Health

© 2023 Chen S et al. *JAMA Oncology*.

Expenditures, Labor Participation Rate, Physical Capital, Population, and Saving Rate, Respectively

**eTable 4.** Estimating the Relationship Among Percentage of Economic Loss, the Two Main PCA Components of IHME Data, and the Indicator of High-Income Countries

**eAppendix 5.** Geographic Distribution and Discounted Estimates of Economic Burden of Cancers

**eFigure 5.** Economic Cost of Cancers as a Percentage of Total GDP in 2020–2050

**eTable 5.** Total Macroeconomic Cost, Economic Cost as a Share of GDP in 2020–2050, and Per Capita Economic Cost Attributable to Cancers, by World Bank Region and Country (in 2017 INT$), Using Discount Rates of 2%

**eTable 6.** Total Macroeconomic Burden Attributable to Cancers in 2020–2050 Using a Discount Rate of 0% for 204 Countries, by World Bank Region (in 2017 INT$)

**eTable 7.** Total Macroeconomic Burden Attributable to Cancers in 2020–2050 Using a Discount Rate of 3% for 204 Countries, by World Bank Region (in 2017 INT$)

**eFigure 6.** Cancer Type Responsible for the Most DALYs in 2019 for Each Country (TBL = Tracheal, Bronchus, and Lung)

**eTable 8.** Total Macroeconomic Cost, Economic Cost as a Share of Total GDP in 2020–2050, and Per Capita Economic Cost Attributable to Cancer Mortality and Morbidity, by World Bank Region, by World Bank Income Group, and Globally With Discount Rate of 0%

**eTable 9.** Total Macroeconomic Cost, Economic Cost as a Share of Total GDP in 2020–2050, and Per Capita Economic Cost Attributable to Cancer Mortality and

© 2023 Chen S et al. *JAMA Oncology*.

Morbidity, by World Bank Region, by World Bank Income Group, and Globally With Discount Rate of 3%

**eAppendix 6.** Differences in Macroeconomic Loss and Lifetime Disease Burden (**eTable 10** )

**eTable 10.** Comparison of Macroeconomic Loss and Lifetime Disease Burden by World Bank Region and Country Income Group

**eAppendix 7.** Contribution of Treatment Costs and Human Capital

**eFigure 7.** Contribution of Treatment Costs to the Total Economic Cost of Cancers by Country Income Group and World Bank Region

**eFigure 8.** Contribution of Human Capital to the Total Economic Cost of Cancers by Country Income Group and World Bank Region

**eAppendix 8.** Strengths and Limitations

**eTable 11.** Strengths and Limitations of Our Model

**eReferences**

This supplemental material has been provided by the authors to give readers additional information about their work.

© 2023 Chen S et al. *JAMA Oncology*.

## eAppendix 1. Global health burden of cancer

**Figures S1–S4** show the health burden of cancer. The numbers are based on the Global Burden of Disease Study (2020).[1]

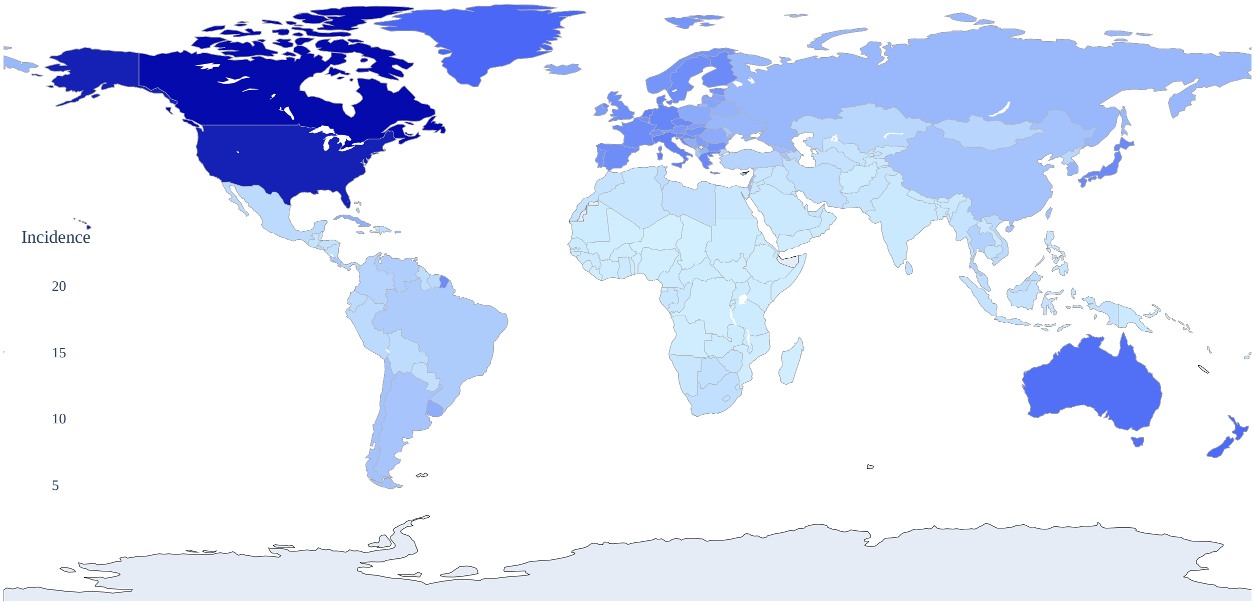


*White areas represent countries with insufficient data

**eFigure 1. All-cancer incidence rate (per 1,000) in 2019**

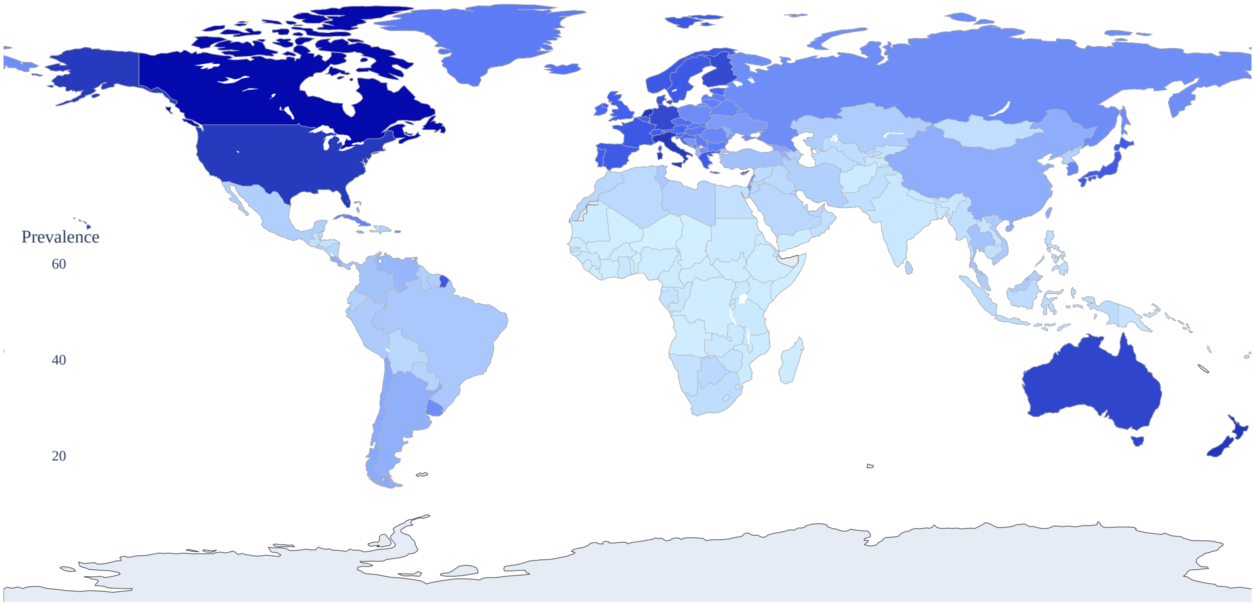


*White areas represent countries with insufficient data

**eFigure 2. All-cancer prevalence rate (per 1,000) in 2019**

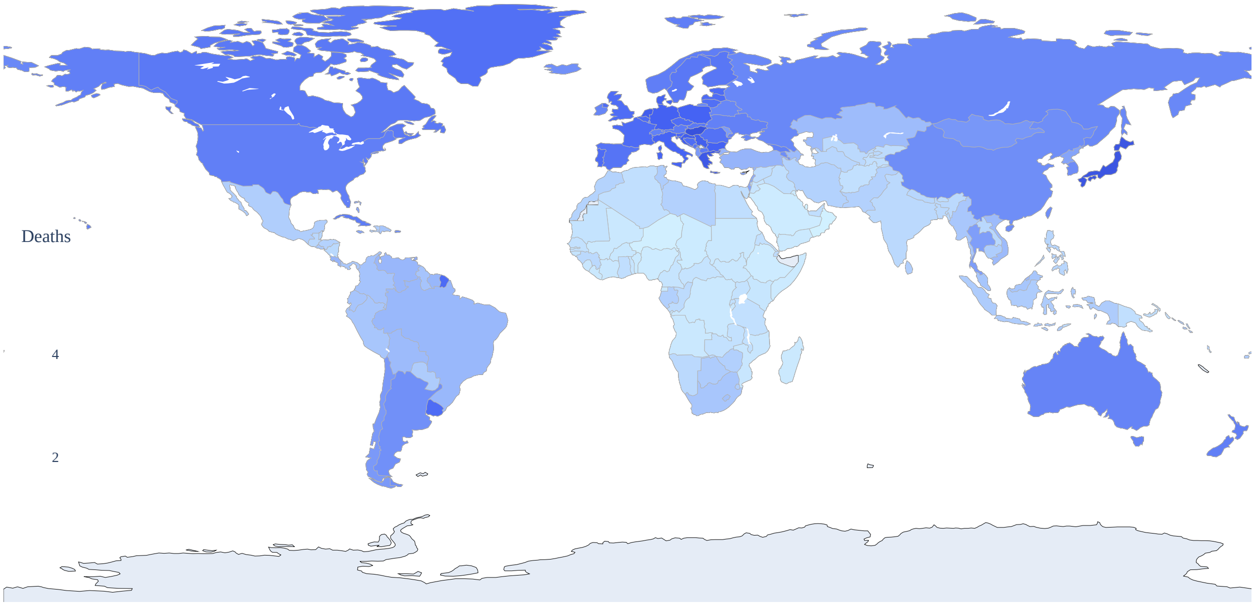


*White areas represent countries with insufficient data

**eFigure 3. All-cancer mortality rate (per 1,000) in 2019**

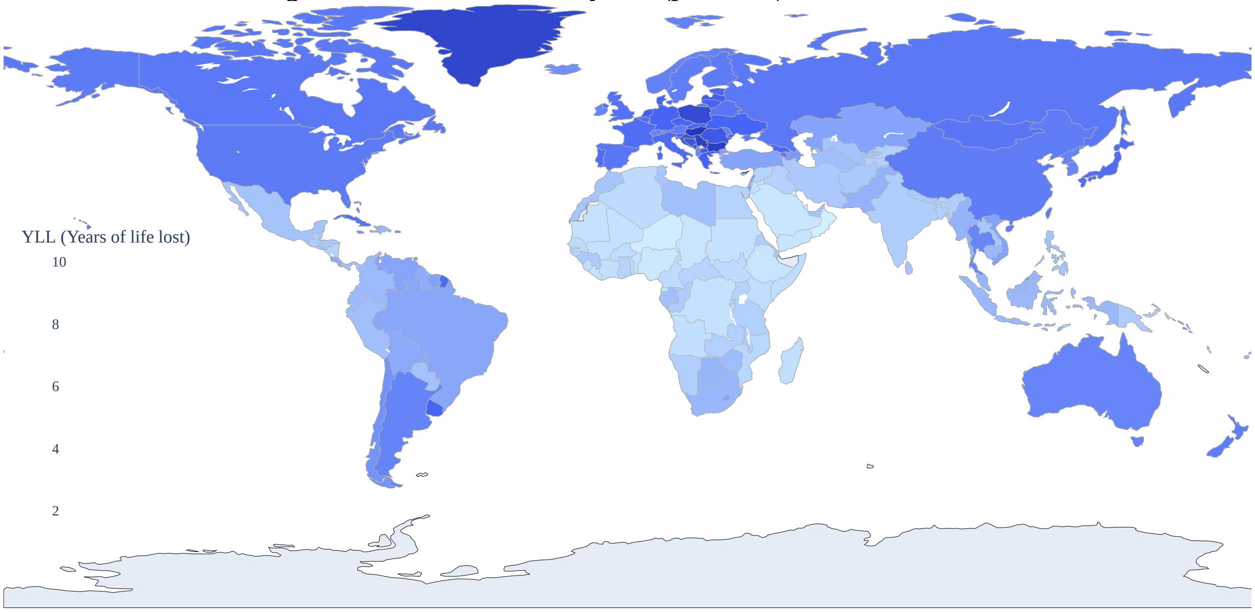


*White areas represent countries with insufficient data

**eFigure 4. Years of life lost to all cancers (per 1,000) in 2019**

## eAppendix 2. Modeling details

We aimed to quantify each type of cancer's impact on economic output through healthcare expenditures and through productivity losses due to mortality and morbidity. For each country and each cancer, we did the following analysis:

**Step 1.** We identified the disease burden of cancer (in terms of mortality, morbidity, and treatment costs).
**Step 2.** We constructed economic projections for two scenarios: a status quo scenario, in which gross domestic product (GDP) is projected to grow based on current estimates and projections of disease prevalence, and a counterfactual scenario, in which cancer prevalence is eliminated from the beginning of

the time frame. The economic projections utilize a macroeconomic production function and can be further decomposed into two parts:

a) Projections of effective labor supply.
b) Projections of physical capital accumulation.

**Step 3.** We calculated the economic loss as the discounted cumulative difference in projected annual GDP between these two scenarios by discount rate.

This detailed model description follows our previous contribution, in which we applied the framework to estimate the economic burden of noncommunicable diseases in China, Japan, and South Korea[2] and in the United States and European countries[3 4] as well as the economic burden of chronic obstructive pulmonary disease and other risk factors.[4-7]

*Production function*

Consider an economy in which time $t = 1,2, \ldots, \infty$ evolves discretely. Building on Lucas,[8] we considered the following production function for this economy:

$$Y_t = A_t K_t^{\alpha} H_t^{1-\alpha}, \tag{1}$$

where $Y_t$ is aggregate output; $A_t$ is the technological level at time $t$, which we assume evolves exogenously; $K_t$ is the physical capital stock (i.e., machines, factory buildings, etc.); and $H_t$ represents aggregate human capital. The parameter $\alpha$ is the elasticity of final output with respect to physical capital. The aggregate production function recognizes that output is not only produced with physical capital and *raw labor* as in the Solow framework,[9] on which the original EPIC model is based,[10] but with *effective labor*, of which health is a crucial determinant.

Physical capital evolves according to

$$K_{t+1} = (1-\delta)K_t + Y_t - C_t - TC_t = (1-\delta)K_t + \gamma_t Y_t \,, \tag{2}$$

where $\delta$ refers to the depreciation rate, $\gamma_t$ refers to the saving rate, $TC_t$ refers to the costs of ongoing treatment of cancer (or the types of cancer under consideration), and $C_t$ refers to the amount of consumption. From Equation (2), it follows that the saving rate is defined as

$$\gamma_t = 1 - \frac{C_t + TC_t}{Y_t}. \tag{3}$$

Note that aggregate output $Y_t$ is used for three purposes: (i) to pay treatment costs $TC_t$ (hospitalization, medication, etc.), (ii) to consume the amount $C_t$, and (iii) to save.

Individuals of age-sex group $(a, s)$ with age $a$ and sex $s$ are endowed with $h_t^{(a,s)}$ units of human capital and supply $\ell_t^{(a,s)}$ units of labor from age 15 up to their retirement. Children younger than 15 and retirees older than the age of $R$ do not work. $R$ varies by country and could correspond to a high age (e.g., some people older than 80 could also be working). In the theoretical derivations, $R$ indicates the upper bound of the summation. In our simulations, we used labor projections data from the International Labour Organization, and positive values for the labor force exist for cohorts older than 65. We discretized age less than 65 into 13 groups every five years and discretized all ages older than 65 into 1 group. Thus, we have 28 age-sex groups marked as $G$. Aggregate human capital in the production function (1) is then defined as the sum over the age-sex-time-specific effective labor supply of each age-sex group:

$$H_t = \sum_{(a,s)\in G} h_t^{(a,s)} \ell_t^{(a,s)} N_t^{(a,s)} \,, \tag{4}$$

where $N_t^{(a,s)}$ denotes the number of individuals in age-sex group $(a, s)$. Note that aggregate human capital increases with the number of working-age individuals who live in the economy (i.e., with a higher $N_t = \sum_{(a,s)\in G} N_t^{(a,s)}$), with individual human capital endowment (i.e., with a higher $h_t^{(a,s)}$ for at least one group), and with labor supply (i.e., with a higher $\ell_t^{(a,s)}$ for at least one group).

We followed Mincer[11] and constructed the average human capital for each age-sex group with age $a$ according to an exponential function of education and work experience:

$$h_t^{(a,s)} = \exp\left[\eta_1\left(ys_t^{(a,s)}\right) + \eta_2\left(a - ye_t^{(a,s)} - 5\right) + \eta_3\left(a - ye_t^{(a,s)} - 5\right)^2\right], \tag{5}$$

where $\eta_1$ is the semi-elasticity of human capital with respect to average years of education as given by $ye_t^{(a,s)}$, and $\eta_2$ and $\eta_3$ are the semi-elasticities of human capital with respect to the experience of the workforce $\left(a - ye_t^{(a,s)} - 5\right)$ and the experience of the workforce squared $\left(a - ye_t^{(a,s)} - 5\right)^2$, respectively. Here, we assumed a school entry age of 5 years throughout.

*Impact of cancer on labor supply*

Following Bloom et al.[2] and Chen et al.,[3 5 6 12] the evolution of labor supply in the status quo scenario is given by

$$L_t^{(a,x)} = \ell_t^{(a,s)} N_t^{(a,s)} \text{ with } N_t^{(a,s)} = \left[1 - \sigma_{t-1}^{(a-1,s)}\right] N_{t-1}^{(a-1,s)}, \tag{6}$$

where $\sigma_t^{(a,s)}$ is the overall mortality rate of the age-sex group with age $a$ and sex $s$ at time $t$. Mortality and morbidity reduce effective labor supply. The reduction of the population size $N_t^{(a,s)}$ captures the mortality effect.

Let $\sigma_{r,t}^{(a,s)}$ denote the mortality rate of people in each age-sex group due to cancer and let $\sigma_{-r,t}^{(a,s)}$ be the overall mortality rate due to causes other than cancer. Then we have

$$\left(1 - \sigma_t^{(a,s)}\right) = \left(1 - \sigma_{r,t}^{(a,s)}\right)\left(1 - \sigma_{-r,t}^{(a,s)}\right), \tag{7}$$

Next, we considered the mortality effect of cancer. In general, it reduces labor supply by reducing the population $N_t^{(a,s)}$ (through $\sigma_{r,t}^{(a,s)}$). In the counterfactual case, where cancers are eliminated from time $t = 0$ onward, the evolution of labor supply is defined similarly to Equation (6), but with a different overall mortality rate ($\sigma_{-r,t}^{(a,s)}$ instead of $\sigma_t^{(a,s)}$). For simplicity, we assumed that the number of births is the same in both cases at each point in time $t$.

In the counterfactual scenario, the size of the cohort with age $a$ and sex $s$ at time $t$ ($\bar{N}_t^{(a,s)}$) evolves according to

$$\bar{N}_t^{(a,s)} = \left[1 - \sigma_{-r,t-1}^{(a-1,s)}\right] \bar{N}_{t-1}^{(a-1,s)}, \bar{N}_0^{(a,s)} = N_0^{(a,s)}, \bar{N}_t^{(0,s)} = N_t^{(0,s)}. \tag{8}$$

Following Bloom et al.,[2] the loss of labor due to mortality accumulates over the years according to

$$\bar{N}_t^{(a,s)} = \frac{N_t^{(a,s)}}{\prod_{\tau=0}^{\min\{t,a\}-1}\left[1 - \sigma_{r,t-1-\tau}^{(a-1-\tau,s)}\right]}. \tag{9}$$

The reduction of the labor participation rate $\ell_t^{(a,s)}$ captures the morbidity effect because people with an illness typically reduce their labor supply, either by reducing working hours or by leaving the workforce.

Following Bloom et al.,[2] the labor participation rate in the counterfactual scenario $\bar{\ell}_t^{(a,s)}$ can be calculated as

$$\bar{\ell}_t^{(a,s)} \approx \frac{\ell_t^{(a,s)}}{\prod_{\tau=0}^{\min\{t,a\}-1}\left[1 - p^\tau \sigma_{r,t-1-\tau}^{(a-1-\tau,s)} \xi^{(a-1-\tau,s)}\right]}, \tag{10}$$

where $\xi^{(a,s)}$ measures the size of the morbidity effect relative to the relevant mortality rate, and $p$ is the probability of a patient not recovering from cancer morbidity in each year.

Because the impact of morbidity is hard to estimate directly, we first defined the relative effect for each age-sex group:

$$\xi^{(a,s)} = \frac{\text{loss of labor due to morbidity of group } (a,s)}{\text{loss of labor due to mortality of group } (a,s)}\,. \tag{11}$$

Next, we assumed that the following holds in any given year for each age-sex group:

$$\xi^{(a,s)} = \frac{YLD^{(a,s)}}{YLL^{(a,s)}}\,, \tag{12}$$

where $YLD^{(a,s)}$ represents the years lived with cancer and $YLL^{(a,s)}$ represents the years of life lost due to cancer. Notice that $\xi^{(a,s)}$ can be calculated from the corresponding DALY data reported by the Global Burden of Disease Study.[1]

In sum, by reducing the prevalence of cancer, the *counterfactual scenario* is associated with an increase in labor supply as compared with the *status quo scenario.* We approximated the change in labor supply (at time $t$ for each age-sex group) by

$$\Delta L_t^{(a,s)} \approx \ell_t^{(a,s)} N_t^{(a,s)} \sum_{\tau=0}^{\min\{t,a\}-1} \sigma_{r,t-1-\tau}^{(a-1-\tau,s)}\left[1 + p^\tau \xi^{(a-1-\tau,s)}\right]. \tag{13}$$

For the more general case of a partial reduction in the prevalence of cancer by a factor $\rho$, we obtained the loss of labor for each age-sex group at time $t$ as

$$\Delta L_t^{(a,s)}(\rho) \approx \ell_t^{(a,s)} N_t^{(a,s)} \sum_{\tau=0}^{\min\{t,a\}-1} \rho\sigma_{r,t-1-\tau}^{(a-1-\tau,s)}\left[1 + p^\tau \xi^{(a-1-\tau,s)}\right]. \tag{14}$$

Bloom et al. (2020)[2] provides the detailed mathematical proof.

*Impact of cancer on physical capital accumulation*

Cancers also impede the accumulation of physical capital because savings finance part of the treatment costs. Following Bloom et al.[2] and Chen et al.,[3] physical capital accumulation in the counterfactual scenario can be written as

$$\bar{K}_{t+1} = \bar{\gamma}_t \bar{Y}_t + (1-\delta)\bar{K}_t\,, \tag{15}$$

$$\bar{\gamma}_t \bar{Y}_t = \bar{I}_t = \bar{Y}_t - \bar{C}_t = \gamma_t \bar{Y}_t + \chi TC_t, \tag{16}$$

where an overbar indicates the counterfactual scenario and where $\chi$ is the fraction of the treatment cost that is diverted to savings. The counterfactual saving rate is thus defined by

$$\bar{\gamma}_t = \frac{\gamma_t \bar{Y}_t + \chi TC_t}{\bar{Y}_t}. \quad (17)$$

For more details, see Bloom et al. (2020)[2] and Chen et al. (2018).[3]

Because cancers are assumed to be eliminated in the counterfactual scenario, the resources that were devoted to their treatment can now be used for savings or for consumption. Notice that this creates an income effect that, in reality, could affect the division of households' income between savings and consumption. For tractability, we assumed that aggregate investment consists of two parts in the counterfactual scenario: a fixed share $\gamma_t$ of total output and an additional part from $TC_t$ that would otherwise have been used to pay to treat cancers:

$$\bar{I}_t = \gamma_t \bar{Y}_t + \chi TC_{t,} \quad (18)$$

Similarly, for the case of a partial reduction in cancer prevalence by $\rho$, we have

$$\bar{I}_t = \gamma_t \bar{Y}_t + \rho \chi TC_t. \quad (19)$$

The intuition is that if cancers are partially eliminated, the treatment cost that is diverted to savings should be added back proportionally.

## eAppendix 3. Data description

### *Education*

Age-specific educational attainment data are from the Barro-Lee Educational Attainment Database,[13] which provides educational attainment data by five-year age-sex groups up to 2010. For 2010–2030, no age-specific data are available, but the database provides projections for the population aged 15–64. We approximated the age-specific estimates by assuming that educational attainment for each age-sex group grows at the same rate. Because the Barro-Lee database presents data in five-year intervals, linear interpolation was adopted to extend the estimates for each year. For 2030–2050, we projected educational attainment by assuming the same growth rate as during 2010–2030 for each age and sex group.

### *Mortality/morbidity*

The age-sex-specific mortality and morbidity (measured in years of life lost and years of life lost to disability) of cancers up to 2019 are from the recently updated GBD estimates.[1] To extend the estimates beyond 2019, we assumed that the mortality rate of cancers grows at the same rate as during 2010–2019 for each country. Morbidity estimates were obtained similarly. If the projected mortality rate grew too large (i.e., if it more than doubled the current annual rate in 30 years), we limited the mortality rate's growth rate to 2%.

### *GDP projection*

The GDP estimates (in constant 2017 international dollars or INT$) up to 2020 are from the World Bank database.[14] The GDP growth rates for 2021–2027 are from the International Monetary Fund's World Economic Outlook as of April 2022.[15] We assumed that growth beyond 2027 will be the same as in 2015–2019.

### *Physical capital*

For each country, the physical capital stock (in constant 2017 INT$) was obtained from the Penn World Table projections.[16]

*Labor participation*

For each country, the labor participation rates (by five-year age-sex groups) are from the International Labour Organization database for 2010–2020.[17] For estimates beyond 2020, we estimated the labor participation rates for the five-year age groups using a logistic regression.

*Population*

For each country, the population by five-year age group was obtained from the population dynamics database built by the Department of Economic and Social Affairs (DESA) of the United Nations.[18] For those countries without five-year age group data, we used the total population from DESA to impute the economic burden.

*Saving rate and health expenditure*

We obtained country-specific saving rates and health expenditures from the World Bank database.[19] For the projection, we assumed that the saving rate remains constant (at the average from 2010–2019), while health expenditures (as a percentage of GDP) grow at the same rate as in 2000–2019.

*Parameter values and data sources*

**eTable 1** shows parameter values and data sources in the model, where definitions for parameters are consistent with Bloom et al. (2020)[2] and Chen et al. (2019).[5 12]

**eTable 1. Parameter values and data sources**

| Parameter | **Definition** | Value | Source |
|---|---|---|---|
| | Capital share | Country specific | Penn World Table[20] |
| | Depreciation rate | 0.05 | Grossmann et al. (2013)[21] |
| | Mincer elasticity of education | 0.091 | Psacharopoulos and Patrinos (2018)[22] |
| | First-degree Mincer elasticity of experience | 0.1301 | Heckman et al. (2006)[23] |
| | Second-degree Mincer elasticity of experience | −0.0023 | Heckman et al. (2006)[23] |
| | Fraction of treatment cost financed out of savings | Set as saving rate | World Bank (2022)[19] |

*Treatment costs*

Prior studies of cancer treatment costs for the United States and other countries have typically focused on the patient level (rather than the national level) and have been limited to patients who actively sought care.[24-26] This makes obtaining national-level cost estimates difficult because many people with cancer are either not diagnosed or do not actively seek care. In addition, available estimates typically fail to consider comorbidities and vary substantially. In our study, total treatment costs for each cancer in the United States are based on results from Dieleman et al. (2020),[27] who systematically estimated national-level spending on private insurance, public insurance, and out-of-pocket payments for different conditions after considering comorbidities. Their spending estimates amounted to 4.57% of the total health expenditures in the United States that year ($123.8 billion / $2705.6 billion, in constant 2016 US dollars). **eTable 2** shows the treatment costs for 29 cancers in the United States.

We calculated the country-level cancer-related treatment costs for the countries with data and extrapolated costs for the countries without data, assuming that the per case treatment cost for cancer was proportional to the health expenditure per capita of the country, as previous studies have assumed.[12 28 29] The intuition here is that health expenditure per capita could be a good metric of the cost of treating a certain disease (cancer for example) across countries. This is an approximate estimate for tractability due to lack of data. Under this assumption, share of cancer-related treatment costs out of all health expenditures (all treatment costs) can be calculated via the cancer prevalence rate. Specifically, we used the U.S. data as the base share of cancer-related treatment costs out of all health expenditures and scaled this figure by the ratio of cancer prevalence between other countries and the United States. For years after 2010, we assumed that the treatment costs of cancers grow at the same rate as per capita health expenditures for each country.

**eTable 2. Treatment costs (medium, lower bound, and upper bound) and the share of all treatment cost for all 29 cancers in the United States**

| Disease | Treatment cost (billions of 2016 US dollars) | Treatment cost_lower (billions of 2016 US dollars) | Treatment cost_upper (billions of 2016 US dollars) | Share of all treatment cost (%) |
|---|---|---|---|---|
| **All cancer** | 123.8 | 114.9 | 132.8 | 4.575695 |
| **Non-melanoma skin cancer** | 21.6 | 17.7 | 27.5 | 0.798344 |
| **Other neoplasms** | 13.2 | 11 | 15.6 | 0.487877 |
| **Leukemia** | 12 | 10.7 | 13.6 | 0.443525 |
| **Non-Hodgkin lymphoma** | 11.8 | 10.4 | 13.4 | 0.436133 |
| **Colon and rectum cancer** | 10.5 | 9.3 | 11.7 | 0.388084 |
| **Tracheal, bronchus, and lung cancer** | 7.3 | 6.3 | 8.4 | 0.269811 |
| **Multiple myeloma** | 7.1 | 6.1 | 8.5 | 0.262419 |
| **Brain and central nervous system cancer** | 6.8 | 6 | 7.6 | 0.251331 |
| **Prostate cancer** | 5.94 | 5.4 | 6.6 | 0.219545 |
| **Breast cancer** | 4.5 | 3.6 | 5.5 | 0.166322 |
| **Kidney cancer** | 3.4 | 2.9 | 3.9 | 0.125665 |
| **Bladder cancer** | 2.6 | 2.3 | 2.9 | 0.096097 |
| **Pancreatic cancer** | 2.5 | 2.2 | 2.8 | 0.092401 |
| **Liver cancer** | 1.7 | 1.5 | 2.1 | 0.062833 |
| **Ovarian cancer** | 1.6 | 1.3 | 2 | 0.059137 |
| **Stomach cancer** | 1.5 | 1.2 | 2 | 0.055441 |
| **Lip and oral cavity cancer** | 1.4 | 1.1 | 1.6 | 0.051745 |
| **Malignant skin melanoma** | 1.4 | 1.3 | 1.6 | 0.051745 |
| **Esophageal cancer** | 1.1 | 0.881 | 1.3 | 0.040656 |
| **Uterine cancer** | 1.1 | 0.924 | 1.2 | 0.040656 |
| **Thyroid cancer** | 0.891 | 0.764 | 1 | 0.032932 |
| **Hodgkin lymphoma** | 0.851 | 0.575 | 1.2 | 0.031453 |
| **Cervical cancer** | 0.81 | 0.653 | 0.97 | 0.029938 |
| **Larynx cancer** | 0.787 | 0.602 | 0.965 | 0.029088 |
| **Other pharynx cancer** | 0.685 | 0.497 | 0.794 | 0.025318 |
| **Testicular cancer** | 0.394 | 0.252 | 0.71 | 0.014562 |
| **Gallbladder and biliary tract cancer** | 0.358 | 0.311 | 0.411 | 0.013232 |
| **Nasopharynx cancer** | 0.06 | 0.049 | 0.072 | 0.00222 |

## eAppendix 4. Imputation

**eTable 3** describes some data lacking for 60 countries**.**

**eTable 3. Missing data for 60 countries; the columns represent regions, country code, country name, GDP, treatment costs, education, health expenditures, labor participation rate, physical capital, population, and saving rate, respectively**

| Region | Code | World Bank Country | GDP | TC | EDU | HEP C | Lab | Cap | Pop | Save |
|---|---|---|---|---|---|---|---|---|---|---|
| **East Asia & Pacific** | ASM | American Samoa | X | | | X | X | X | X | X |
| | PRK | Korea, Dem. People's Rep. | X | | X | X | | X | | X |
| | GUM | Guam | X | | | X | | X | | X |
| | KIR | Kiribati | | | | | X | X | | |
| | MHL | Marshall Islands | | | | | X | X | X | |
| | FSM | Micronesia, Fed. Sts. | | | | | X | X | | X |
| | MMR | Myanmar | | | | | | X | | |
| | NRU | Nauru | | | | | X | X | X | X |
| | MNP | Northern Mariana Islands | X | | | X | X | X | X | X |
| | PLW | Palau | | | | | X | X | X | X |
| | PNG | Papua New Guinea | | | | | | X | | X |
| | WSM | Samoa | | | | | | X | | X |
| | SLB | Solomon Islands | | | | | | X | | |
| | TWN | Taiwan (Province of China) | X | | | X | | | | X |
| | TLS | Timor-Leste | | | | | | X | | |
| | TON | Tonga | | | | | | X | | |
| | TUV | Tuvalu | | | | | X | X | X | X |
| | VUT | Vanuatu | | | | | | X | | |
| **Europe & Central Asia** | AND | Andorra | X | | | X | X | X | X | X |
| | ROU | Romania | | | | X | | | | |
| | GRL | Greenland | X | | | X | X | X | X | X |
| | MCO | Monaco | X | | | X | X | X | X | X |
| | SMR | San Marino | | | | | X | X | X | X |
| | TKM | Turkmenistan | | | | | | | | X |
| **Latin America & Caribbean** | ATG | Antigua and Barbuda | | | | | X | | | |
| | CUB | Cuba | X | | | X | | X | | X |
| | DMA | Dominica | | | | | X | | X | |
| | GRD | Grenada | | | | | X | | | X |
| | GUY | Guyana | | | | | | X | | X |
| | HTI | Haiti | | | | | | X | | |
| | NIC | Nicaragua | | | | | | X | | |
| | PRI | Puerto Rico | | | | X | | X | | X |
| | KNA | St. Kitts and Nevis | | | | | X | | X | X |
| | LCA | St. Lucia | | | | | | | | X |
| | VCT | St. Vincent and the Grenadines | | | | | | | | X |
| | TTO | Trinidad and Tobago | | | | | | | | X |
| | VIR | Virgin Islands (U.S.) | X | | | X | | X | | X |

| Region | Code | World Bank Country | GDP | TC | EDU | HEP C | Lab | Cap | Pop | Save |
|---|---|---|---|---|---|---|---|---|---|---|
| | VEN | Venezuela, RB | X | | | X | | | | |
| **Middle East & North Africa** | DZA | Algeria | | | | | | X | | |
| | IRN | Iran, Islamic Rep. | | | | | | | | X |
| | LBY | Libya | | | | | | X | | X |
| | SYR | Syrian Arab Republic | X | | | X | | | | |
| | ARE | United Arab Emirates | | | | | | X | | X |
| | YEM | Yemen, Rep. | X | | | X | | | | X |
| **North America** | BMU | Bermuda | X | | | X | X | | X | |
| **South Asia** | AFG | Afghanistan | | | | | | X | | X |
| **Sub-Saharan Africa** | CAF | Central African Republic | | | | | | | | X |
| | TCD | Chad | | | | | | | | X |
| | GNQ | Equatorial Guinea | | | | | | | | X |
| | ERI | Eritrea | X | | | X | | X | | X |
| | LBR | Liberia | | | | | | | | X |
| | MWI | Malawi | | | | | | | | X |
| | STP | Sao Tome and Principe | | | | | | | | X |
| | SYC | Seychelles | | | X | | X | X | | |
| | SOM | Somalia | | | | X | | X | | X |
| | SSD | South Sudan | X | | | X | | X | | |
| **Others** | COK | Cook Islands | X | | X | X | X | X | X | X |
| | NIU | Niue | X | | X | X | X | X | X | X |
| | PSE | Palestine | X | | X | X | | X | | |
| | TKL | Tokelau | X | | X | X | X | X | X | X |

For the 60 countries and territories with incomplete data (mostly on education, physical capital, and the saving rate in **eTable 3**) but reliable data for GDP and DALYs, we used a linear projection to approximate the economic burden of cancer. We found that the ratio of economic burden to GDP is highly correlated to DALYs. Thus, we imputed economic burden results based on an ordinary least squares linear regression of the relationship between GDP and DALYs. For the imputation, we then used the formula

$$Y_{imputed} = \beta_0 + \beta_1 \text{DALYs}, \tag{20}$$

where and are the parameter estimates for the intercept and the slope obtained in the linear regression.

**eTable 4** shows the regression coefficients with p-values. All p-values of the coefficient of DALYs are less than 0.0001. The average number of DALYs is 3,233, which means the constant value is far less than the coefficient of DALYs multiplying DALYs. Therefore, the large p-value of the coefficient of constant value is also acceptable for some cancers. To improve the fitting result, we replaced DALYs with all healthy data such as the mortality, incidence, and prevalence rates; DALYs; years of life lost to disability; and years of life lost for each cancer. Due to the multicollinearity of these IHME variables, we performed principal component analysis (PCA) and set the two main components of the PCA results as independent variables. We got a better fit for the linear regression for almost all cancers except non-melanoma skin cancer. In this paper, we used the primary results for almost all cancers except other neoplasms using PCA-refined methods (R-squared is 0.7).

**eTable 4. Estimating the relationship among percentage of economic loss, the two main PCA components of IHME data, and the indicator of high-income countries**

| Disease | Coefficient of constant value $\beta_0$ | Coefficient of DALYs $\beta_1$ | p-value | R-squared |
|---|---|---|---|---|
| **Bladder cancer** | 1.24E-05 | 9.10E-07 | 0.000 | 0.66292636 |
| **Brain and central nervous system cancer** | -7.84E-06 | 2.31E-06 | 0.000 | 0.71212245 |
| **Breast cancer** | 6.89E-05 | 1.29E-06 | 0.000 | 0.53856703 |
| **Cervical cancer** | -4.74E-06 | 2.04E-06 | 0.000 | 0.82542384 |
| **Colon and rectum cancer** | 7.38E-05 | 1.04E-06 | 0.000 | 0.71032899 |
| **Esophageal cancer** | 2.09E-05 | 1.16E-06 | 0.000 | 0.72638618 |
| **Gallbladder and biliary tract cancer** | 3.16E-06 | 9.32E-07 | 0.000 | 0.80129833 |
| **Hodgkin lymphoma** | 5.50E-06 | 2.32E-06 | 0.000 | 0.68511449 |
| **Kidney cancer** | 1.12E-05 | 1.18E-06 | 0.000 | 0.76900808 |
| **Larynx cancer** | 3.81E-07 | 1.50E-06 | 0.000 | 0.77727263 |
| **Leukemia** | 5.39E-05 | 1.70E-06 | 0.000 | 0.45658393 |
| **Lip and oral cavity cancer** | -6.14E-06 | 1.83E-06 | 0.000 | 0.75992021 |
| **Liver cancer** | 2.71E-06 | 1.45E-06 | 0.000 | 0.86422094 |
| **Malignant skin melanoma** | 2.32E-06 | 1.84E-06 | 0.000 | 0.9161329 |
| **Mesothelioma** | 5.28E-06 | 7.48E-07 | 0.000 | 0.71878609 |
| **Multiple myeloma** | 1.99E-06 | 1.41E-06 | 0.000 | 0.80181043 |
| **Nasopharynx cancer** | -3.55E-06 | 2.43E-06 | 0.000 | 0.94475422 |
| **Non-Hodgkin lymphoma** | 8.68E-06 | 1.88E-06 | 0.000 | 0.7117133 |
| **Non-melanoma skin cancer (with no PCA)** | -2.85E-05 | 3.60E-06 | 0.000 | 0.78063085 |
| **Other neoplasms** | 0.00011222 | 4.83E-06 | 0.000 | 0.2748814 |
| **Other pharynx cancer** | -3.10E-08 | 1.74E-06 | 0.000 | 0.83323188 |
| **Ovarian cancer** | 3.30E-06 | 1.38E-06 | 0.000 | 0.6783696 |
| **Pancreatic cancer** | 2.51E-05 | 9.73E-07 | 0.000 | 0.71352724 |
| **Prostate cancer** | 6.48E-06 | 6.66E-07 | 0.000 | 0.61454087 |
| **Stomach cancer** | 4.18E-05 | 1.19E-06 | 0.000 | 0.67103976 |
| **Testicular cancer** | 1.95E-06 | 3.02E-06 | 0.000 | 0.81170393 |
| **Thyroid cancer** | 2.38E-06 | 1.47E-06 | 0.000 | 0.66716852 |
| **Tracheal, bronchus, and lung cancer** | -2.91E-06 | 1.10E-06 | 0.000 | 0.67689097 |
| **Uterine cancer** | 1.24E-05 | 9.10E-07 | 0.000 | 0.66292636 |

We then imputed the percentage of the economic loss in total GDP using the coefficients from the regression and calculated the economic loss for the 60 countries with incomplete data as listed in **eTable 3**. The results are merged into the main paper.

## eAppendix 5. Geographic distribution and discounted estimates of economic burden of cancers

**eFigure 5** shows the geographical distribution of the economic burden of cancer in percentage terms.

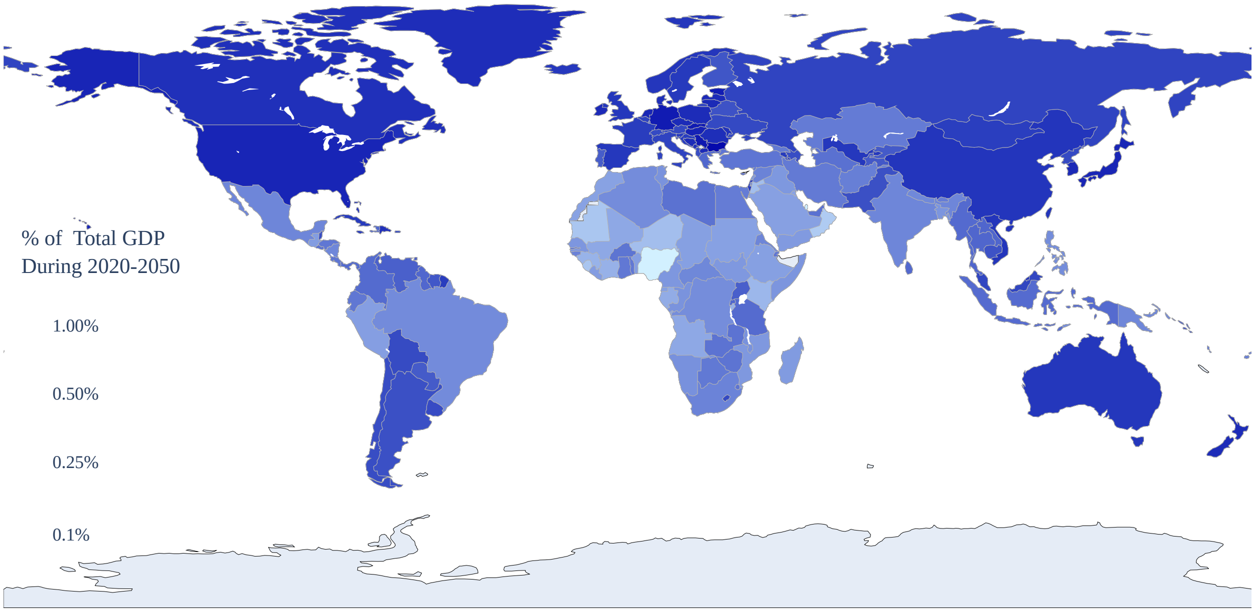


**eFigure 5. Economic cost of cancers as a percentage of total GDP in 2020–2050**

**Tables S5–S7** show the total discounted economic burden of cancers in 2020–2050 for each country, by World Bank region, and by World Bank income group, using discount rates of 2%, 0%, and 3%.

**eTable 5. Total macroeconomic cost, economic cost as a share of GDP in 2020–2050, and per capita economic cost attributable to cancers, by World Bank region and country (in 2017 INT$), using discount rates of 2%**

| Region | World Bank Country | Economic cost in millions of 2017 INT$ (lower and upper bound) | Percentage of total GDP in 2020–2050 (lower and upper bound) | Per capita loss in 2017 INT$ (lower and upper bound) |
|---|---|---|---|---|
| **East Asia & Pacific** | American Samoa* | 112(67-180) | 0.477(0.286-0.763) | 2,064(1,237-3,300) |
| **East Asia & Pacific** | Australia | 246,962(180,343-345,480) | 0.607(0.443-0.849) | 8,424(6,152-11,785) |
| **East Asia & Pacific** | Brunei Darussalam | 5,442(3,184-8,684) | 0.723(0.423-1.154) | 11,438(6,693-18,251) |
| **East Asia & Pacific** | Cambodia | 18,768(10,624-31,028) | 0.466(0.264-0.770) | 960(543-1,587) |
| **East Asia & Pacific** | China | 6,089,513(4,116,179-8,545,632) | 0.621(0.420-0.872) | 4,208(2,845-5,906) |
| **East Asia & Pacific** | Fiji | 991(476-1,789) | 0.305(0.147-0.551) | 998(479-1,802) |
| **East Asia & Pacific** | Guam* | 1,084(666-1,683) | 0.523(0.321-0.811) | 5,892(3,619-9,144) |
| **East Asia & Pacific** | Indonesia | 534,305(327,951-858,682) | 0.365(0.224-0.587) | 1,740(1,068-2,796) |
| **East Asia & Pacific** | Japan | 967,620(838,479-1,090,630) | 0.751(0.650-0.846) | 8,283(7,178-9,336) |
| **East Asia & Pacific** | Kiribati* | 49(28-81) | 0.567(0.328-0.933) | 331(191-545) |
| **East Asia & Pacific** | Korea, Dem. People's Rep.* | 7,575(4,433-12,286) | 0.529(0.310-0.858) | 285(167-462) |
| **East Asia & Pacific** | Korea, Rep. | 500,271(368,524-695,568) | 0.680(0.501-0.946) | 9,991(7,360-13,891) |
| **East Asia & Pacific** | Lao PDR | 10,815(5,850-18,113) | 0.388(0.210-0.649) | 1,267(685-2,121) |
| **East Asia & Pacific** | Malaysia | 175,758(83,966-318,020) | 0.479(0.229-0.867) | 4,731(2,260-8,560) |
| **East Asia & Pacific** | Marshall Islands* | 33(18-57) | 0.440(0.235-0.763) | 483(258-837) |
| **East Asia & Pacific** | Micronesia, Fed. Sts.* | 54(22-104) | 0.515(0.206-0.986) | 418(167-801) |
| **East Asia & Pacific** | Mongolia | 9,191(4,830-16,562) | 0.566(0.297-1.020) | 2,361(1,241-4,254) |

| | | | | |
|---|---|---|---|---|
| **East Asia & Pacific** | Myanmar* | 41,063(28,515-60,299) | 0.391(0.271-0.574) | 691(480-1,015) |
| **East Asia & Pacific** | Nauru* | 16(9-26) | 0.375(0.218-0.608) | 1,460(846-2,364) |
| **East Asia & Pacific** | New Zealand | 57,733(42,784-78,652) | 0.755(0.560-1.029) | 10,938(8,106-14,901) |
| **East Asia & Pacific** | Northern Mariana Islands* | 337(207-534) | 0.758(0.465-1.201) | 5,496(3,370-8,705) |
| **East Asia & Pacific** | Palau* | 53(32-86) | 0.748(0.449-1.209) | 2,912(1,748-4,707) |
| **East Asia & Pacific** | Papua New Guinea* | 4,102(2,458-6,680) | 0.304(0.182-0.495) | 354(212-576) |
| **East Asia & Pacific** | Philippines | 196,048(117,732-300,613) | 0.418(0.251-0.642) | 1,521(913-2,332) |
| **East Asia & Pacific** | Samoa* | 128(71-211) | 0.324(0.180-0.533) | 551(305-906) |
| **East Asia & Pacific** | Singapore | 64,796(50,470-84,240) | 0.348(0.271-0.452) | 10,301(8,024-13,393) |
| **East Asia & Pacific** | Solomon Islands* | 347(199-574) | 0.638(0.365-1.055) | 357(204-590) |
| **East Asia & Pacific** | Taiwan (Province of China)* | 289,667(152,896-536,189) | 0.708(0.374-1.310) | 12,271(6,477-22,714) |
| **East Asia & Pacific** | Thailand | 152,912(80,089-275,739) | 0.369(0.193-0.666) | 2,210(1,158-3,986) |
| **East Asia & Pacific** | Timor-Leste* | 452(242-704) | 0.333(0.178-0.519) | 268(143-418) |
| **East Asia & Pacific** | Tonga* | 102(60-168) | 0.505(0.295-0.828) | 849(496-1,392) |
| **East Asia & Pacific** | Tuvalu* | 12(7-20) | 0.452(0.259-0.758) | 852(488-1,430) |
| **East Asia & Pacific** | Vanuatu* | 108(59-177) | 0.363(0.198-0.599) | 252(138-415) |
| **East Asia & Pacific** | Vietnam | 327,797(173,817-573,518) | 0.651(0.345-1.139) | 3,114(1,651-5,448) |
| **Europe & Central Asia** | Albania | 8,768(4,484-15,429) | 0.657(0.336-1.156) | 3,254(1,664-5,727) |
| **Europe & Central Asia** | Andorra* | 927(575-1,419) | 0.778(0.483-1.191) | 11,948(7,414-18,283) |
| **Europe & Central Asia** | Armenia | 8,367(5,531-12,772) | 0.554(0.366-0.845) | 2,861(1,892-4,368) |
| **Europe & Central Asia** | Austria | 62,132(48,969-80,737) | 0.465(0.366-0.604) | 6,783(5,346-8,814) |
| **Europe & Central Asia** | Azerbaijan | 17,711(9,595-30,403) | 0.469(0.254-0.805) | 1,637(887-2,810) |
| **Europe & Central Asia** | Belarus | 20,164(10,289-39,750) | 0.481(0.246-0.949) | 2,219(1,132-4,375) |
| **Europe & Central Asia** | Belgium | 81,499(59,111-114,154) | 0.510(0.370-0.715) | 6,801(4,933-9,526) |
| **Europe & Central Asia** | Bosnia and Herzegovina | 12,689(7,576-20,285) | 0.766(0.458-1.225) | 4,211(2,514-6,732) |
| **Europe & Central Asia** | Bulgaria | 76,830(42,006-134,380) | 1.416(0.774-2.477) | 12,490(6,829-21,846) |
| **Europe & Central Asia** | Croatia | 20,065(11,413-35,775) | 0.524(0.298-0.934) | 5,354(3,045-9,545) |
| **Europe & Central Asia** | Cyprus | 5,108(3,583-7,310) | 0.355(0.249-0.508) | 3,946(2,767-5,647) |
| **Europe & Central Asia** | Czech Republic | 104,503(69,200-163,335) | 0.713(0.472-1.115) | 9,794(6,485-15,307) |
| **Europe & Central Asia** | Denmark | 62,083(44,902-87,762) | 0.610(0.441-0.862) | 10,249(7,413-14,488) |
| **Europe & Central Asia** | Estonia | 17,844(10,318-30,196) | 1.018(0.588-1.722) | 14,300(8,269-24,197) |
| **Europe & Central Asia** | Finland | 32,496(24,365-44,579) | 0.435(0.326-0.597) | 5,854(4,389-8,030) |
| **Europe & Central Asia** | France | 478,125(330,702-697,800) | 0.600(0.415-0.876) | 7,143(4,940-10,425) |
| **Europe & Central Asia** | Georgia | 6,870(4,398-10,104) | 0.313(0.201-0.461) | 1,824(1,167-2,682) |
| **Europe & Central Asia** | Germany | 1,215,479(896,484-1,661,940) | 1.014(0.748-1.386) | 14,749(10,878-20,166) |
| **Europe & Central Asia** | Greece | 28,944(21,728-38,561) | 0.374(0.281-0.499) | 2,978(2,236-3,968) |

| | | | | |
|---|---|---|---|---|
| **Europe & Central Asia** | Greenland* | 677(393-1,120) | 0.784(0.455-1.297) | 12,098(7,025-20,008) |
| **Europe & Central Asia** | Hungary | 105,943(63,857-174,025) | 0.909(0.548-1.493) | 11,634(7,013-19,111) |
| **Europe & Central Asia** | Iceland | 4,240(2,926-5,953) | 0.608(0.420-0.854) | 11,654(8,043-16,361) |
| **Europe & Central Asia** | Ireland | 289,186(213,306-405,025) | 0.745(0.549-1.043) | 54,009(39,837-75,643) |
| **Europe & Central Asia** | Italy | 302,171(254,813-353,700) | 0.503(0.424-0.589) | 5,216(4,399-6,106) |
| **Europe & Central Asia** | Kazakhstan | 50,474(33,300-76,262) | 0.304(0.201-0.460) | 2,349(1,550-3,549) |
| **Europe & Central Asia** | Kyrgyz Republic | 4,797(2,993-7,289) | 0.419(0.262-0.637) | 610(381-927) |
| **Europe & Central Asia** | Latvia | 14,933(7,696-28,416) | 0.761(0.392-1.449) | 8,995(4,636-17,116) |
| **Europe & Central Asia** | Lithuania | 28,538(18,237-44,783) | 0.762(0.487-1.195) | 11,924(7,620-18,712) |
| **Europe & Central Asia** | Luxembourg | 7,521(5,465-10,391) | 0.315(0.229-0.435) | 10,517(7,642-14,531) |
| **Europe & Central Asia** | Moldova | 7,448(4,800-11,024) | 0.611(0.394-0.904) | 1,989(1,282-2,943) |
| **Europe & Central Asia** | Monaco* | 3,648(2,380-5,560) | 1.328(0.867-2.025) | 85,230(55,612-129,918) |
| **Europe & Central Asia** | Montenegro | 4,420(2,686-6,947) | 1.091(0.663-1.714) | 7,191(4,370-11,301) |
| **Europe & Central Asia** | Netherlands | 228,621(168,484-308,934) | 0.792(0.584-1.071) | 13,163(9,701-17,787) |
| **Europe & Central Asia** | North Macedonia | 8,782(4,634-15,843) | 0.798(0.421-1.439) | 4,395(2,319-7,929) |
| **Europe & Central Asia** | Norway | 58,533(51,204-67,653) | 0.579(0.507-0.670) | 9,667(8,456-11,173) |
| **Europe & Central Asia** | Poland | 365,913(218,836-622,791) | 0.744(0.445-1.266) | 10,174(6,085-17,317) |
| **Europe & Central Asia** | Portugal | 40,923(28,362-58,297) | 0.408(0.283-0.581) | 4,215(2,921-6,004) |
| **Europe & Central Asia** | Romania* | 203,881(123,154-323,060) | 0.901(0.544-1.427) | 11,462(6,924-18,162) |
| **Europe & Central Asia** | Russian Federation | 459,222(303,005-681,690) | 0.499(0.329-0.741) | 3,253(2,147-4,830) |
| **Europe & Central Asia** | San Marino* | 488(270-850) | 0.858(0.475-1.493) | 14,277(7,895-24,835) |
| **Europe & Central Asia** | Serbia | 51,592(28,288-93,193) | 1.073(0.588-1.938) | 6,489(3,558-11,722) |
| **Europe & Central Asia** | Slovak Republic | 58,788(30,845-113,845) | 1.012(0.531-1.959) | 11,123(5,836-21,540) |
| **Europe & Central Asia** | Slovenia | 15,543(8,820-28,278) | 0.543(0.308-0.989) | 7,675(4,355-13,963) |
| **Europe & Central Asia** | Spain | 277,921(200,988-383,951) | 0.522(0.378-0.721) | 6,096(4,408-8,422) |
| **Europe & Central Asia** | Sweden | 88,116(72,564-109,271) | 0.519(0.427-0.643) | 8,160(6,720-10,119) |
| **Europe & Central Asia** | Switzerland | 108,345(78,021-151,404) | 0.626(0.451-0.874) | 11,611(8,362-16,226) |
| **Europe & Central Asia** | Tajikistan | 8,285(5,057-13,323) | 0.411(0.251-0.661) | 649(396-1,044) |
| **Europe & Central Asia** | Turkey | 328,237(198,361-532,155) | 0.322(0.195-0.523) | 3,590(2,169-5,820) |
| **Europe & Central Asia** | Turkmenistan* | 9,551(5,447-16,092) | 0.376(0.215-0.634) | 1,351(770-2,276) |
| **Europe & Central Asia** | Ukraine | 11,136(6,610-17,531) | 0.383(0.227-0.603) | 282(168-444) |
| **Europe & Central Asia** | United Kingdom | 508,397(445,889-578,368) | 0.610(0.535-0.693) | 7,125(6,249-8,105) |
| **Europe & Central Asia** | Uzbekistan | 81,388(48,664-124,419) | 0.622(0.372-0.950) | 2,097(1,254-3,206) |
| **Latin America & Caribbean** | Antigua and Barbuda* | 309(206-448) | 0.506(0.338-0.735) | 2,907(1,942-4,220) |

| | | | | |
|---|---|---|---|---|
| **Latin America & Caribbean** | Argentina | 106,110(75,871-147,935) | 0.463(0.331-0.645) | 2,101(1,502-2,930) |
| **Latin America & Caribbean** | Bahamas, The | 2,149(1,284-3,401) | 0.659(0.394-1.042) | 4,931(2,947-7,805) |
| **Latin America & Caribbean** | Barbados | 750(453-1,173) | 0.778(0.470-1.217) | 2,619(1,582-4,095) |
| **Latin America & Caribbean** | Belize | 324(216-462) | 0.479(0.319-0.682) | 658(438-936) |
| **Latin America & Caribbean** | Bolivia | 16,525(8,819-27,574) | 0.493(0.263-0.823) | 1,189(635-1,985) |
| **Latin America & Caribbean** | Brazil | 191,810(162,924-228,415) | 0.263(0.223-0.313) | 853(724-1,016) |
| **Latin America & Caribbean** | Chile | 61,411(44,458-84,818) | 0.449(0.325-0.621) | 3,102(2,246-4,284) |
| **Latin America & Caribbean** | Colombia | 91,451(47,597-165,683) | 0.388(0.202-0.702) | 1,691(880-3,063) |
| **Latin America & Caribbean** | Costa Rica | 19,560(10,655-33,160) | 0.530(0.288-0.898) | 3,531(1,923-5,986) |
| **Latin America & Caribbean** | Cuba* | 38,006(22,126-60,900) | 0.775(0.451-1.242) | 3,488(2,031-5,589) |
| **Latin America & Caribbean** | Dominica* | 155(97-244) | 0.747(0.464-1.174) | 2,145(1,334-3,372) |
| **Latin America & Caribbean** | Dominican Republic | 63,255(27,352-121,097) | 0.660(0.285-1.263) | 5,263(2,276-10,075) |
| **Latin America & Caribbean** | Ecuador | 13,837(8,479-22,469) | 0.287(0.176-0.467) | 668(410-1,085) |
| **Latin America & Caribbean** | El Salvador | 6,082(2,845-11,226) | 0.373(0.174-0.688) | 893(417-1,648) |
| **Latin America & Caribbean** | Grenada* | 351(246-493) | 0.629(0.440-0.884) | 3,039(2,126-4,267) |
| **Latin America & Caribbean** | Guatemala | 11,518(7,549-17,380) | 0.213(0.139-0.321) | 509(333-767) |
| **Latin America & Caribbean** | Guyana* | 6,883(3,837-11,659) | 0.423(0.236-0.716) | 8,374(4,669-14,185) |
| **Latin America & Caribbean** | Haiti* | 3,562(1,873-6,284) | 0.408(0.214-0.720) | 268(141-473) |
| **Latin America & Caribbean** | Honduras | 6,308(3,126-11,444) | 0.322(0.160-0.584) | 524(260-950) |
| **Latin America & Caribbean** | Jamaica | 4,166(2,138-7,155) | 0.610(0.313-1.047) | 1,378(707-2,367) |
| **Latin America & Caribbean** | Mexico | 177,506(123,243-244,700) | 0.269(0.187-0.371) | 1,229(853-1,694) |
| **Latin America & Caribbean** | Nicaragua* | 3,053(1,840-4,902) | 0.276(0.166-0.444) | 398(240-638) |
| **Latin America & Caribbean** | Panama | 16,287(9,488-27,485) | 0.358(0.208-0.604) | 3,158(1,840-5,329) |
| **Latin America & Caribbean** | Paraguay | 12,681(6,571-22,284) | 0.411(0.213-0.722) | 1,540(798-2,706) |
| **Latin America & Caribbean** | Peru | 31,572(17,701-57,072) | 0.247(0.138-0.446) | 850(477-1,537) |
| **Latin America & Caribbean** | Puerto Rico* | 10,983(6,164-19,271) | 0.494(0.277-0.866) | 3,994(2,242-7,009) |
| **Latin America & Caribbean** | St. Kitts and Nevis* | 224(135-346) | 0.586(0.352-0.904) | 4,015(2,412-6,188) |
| **Latin America & Caribbean** | St. Lucia* | 415(287-589) | 0.670(0.463-0.950) | 2,224(1,536-3,151) |
| **Latin America & Caribbean** | St. Vincent and the Grenadines* | 297(211-410) | 0.700(0.498-0.966) | 2,648(1,883-3,657) |
| **Latin America & Caribbean** | Suriname | 861(474-1,409) | 0.435(0.239-0.712) | 1,337(735-2,188) |
| **Latin America & Caribbean** | Trinidad and Tobago* | 3,307(1,689-6,178) | 0.459(0.235-0.858) | 2,372(1,212-4,431) |
| **Latin America & Caribbean** | Uruguay | 9,666(6,900-13,431) | 0.465(0.332-0.646) | 2,696(1,924-3,746) |
| **Latin America & Caribbean** | Venezuela, RB* | 47,548(23,121-88,472) | 0.494(0.240-0.919) | 1,393(677-2,592) |
| **Latin America & Caribbean** | Virgin Islands (U.S.)* | 1,157(735-1,811) | 0.835(0.530-1.307) | 11,939(7,580-18,685) |

| | | | | |
|---|---|---|---|---|
| **Middle East & North Africa** | Algeria* | 32,632(19,385-52,876) | 0.236(0.140-0.382) | 617(367-1,000) |
| **Middle East & North Africa** | Bahrain | 3,118(2,184-4,617) | 0.135(0.094-0.200) | 1,500(1,050-2,221) |
| **Middle East & North Africa** | Djibouti | 1,019(478-1,992) | 0.325(0.153-0.636) | 878(412-1,717) |
| **Middle East & North Africa** | Egypt, Arab Rep. | 178,444(91,029-339,758) | 0.303(0.155-0.577) | 1,364(696-2,597) |
| **Middle East & North Africa** | Iran, Islamic Rep.* | 109,969(81,492-135,232) | 0.346(0.256-0.425) | 1,157(857-1,422) |
| **Middle East & North Africa** | Iraq | 31,280(20,617-47,774) | 0.223(0.147-0.341) | 564(372-862) |
| **Middle East & North Africa** | Israel | 84,137(60,088-118,060) | 0.590(0.421-0.827) | 7,888(5,633-11,068) |
| **Middle East & North Africa** | Jordan | 5,211(3,076-8,633) | 0.164(0.097-0.272) | 459(271-760) |
| **Middle East & North Africa** | Kuwait | 9,273(7,233-12,314) | 0.188(0.146-0.249) | 1,885(1,470-2,503) |
| **Middle East & North Africa** | Lebanon | 1,014(554-1,742) | 0.228(0.125-0.391) | 159(87-272) |
| **Middle East & North Africa** | Libya* | 36,565(20,018-63,480) | 0.357(0.195-0.620) | 4,669(2,556-8,106) |
| **Middle East & North Africa** | Malta | 7,040(5,141-9,655) | 0.650(0.474-0.891) | 15,942(11,642-21,863) |
| **Middle East & North Africa** | Morocco | 18,427(11,219-30,636) | 0.205(0.125-0.341) | 437(266-726) |
| **Middle East & North Africa** | Oman | 6,459(3,244-13,031) | 0.137(0.069-0.277) | 1,052(528-2,123) |
| **Middle East & North Africa** | Qatar | 7,024(5,025-10,542) | 0.097(0.070-0.146) | 2,038(1,458-3,058) |
| **Middle East & North Africa** | Saudi Arabia | 94,838(64,221-145,289) | 0.199(0.135-0.305) | 2,338(1,583-3,581) |
| **Middle East & North Africa** | Syrian Arab Republic* | 5,251(3,075-8,635) | 0.292(0.171-0.480) | 191(112-314) |
| **Middle East & North Africa** | Tunisia | 7,747(4,929-12,063) | 0.233(0.148-0.363) | 596(379-929) |
| **Middle East & North Africa** | United Arab Emirates* | 81,578(38,143-151,669) | 0.381(0.178-0.708) | 7,766(3,631-14,439) |
| **Middle East & North Africa** | Yemen, Rep.* | 5,998(3,453-9,784) | 0.228(0.131-0.371) | 152(88-248) |
| **North America** | Bermuda* | 1,216(800-1,832) | 0.649(0.427-0.979) | 20,732(13,643-31,240) |
| **North America** | Canada | 363,048(263,104-507,966) | 0.703(0.510-0.984) | 8,638(6,260-12,085) |
| **North America** | United States | 5,253,166(4,654,137-6,079,225) | 0.845(0.749-0.978) | 14,703(13,026-17,015) |
| **South Asia** | Afghanistan* | 5,261(3,007-8,686) | 0.294(0.168-0.486) | 101(57-166) |
| **South Asia** | Bangladesh | 132,218(63,277-261,535) | 0.242(0.116-0.479) | 725(347-1,435) |
| **South Asia** | Bhutan | 1,160(511-2,232) | 0.309(0.136-0.595) | 1,354(597-2,605) |
| **South Asia** | India | 1,420,619(931,130-2,045,243) | 0.282(0.185-0.406) | 924(606-1,330) |
| **South Asia** | Maldives | 637(381-1,019) | 0.192(0.115-0.307) | 1,166(698-1,865) |
| **South Asia** | Nepal | 14,409(7,580-25,467) | 0.271(0.143-0.480) | 429(225-757) |
| **South Asia** | Pakistan | 210,793(123,644-342,220) | 0.481(0.282-0.780) | 748(439-1,215) |
| **South Asia** | Sri Lanka | 36,204(15,823-75,118) | 0.371(0.162-0.770) | 1,648(720-3,419) |
| **Sub-Saharan Africa** | Angola | 10,264(5,445-17,350) | 0.207(0.110-0.350) | 193(103-327) |
| **Sub-Saharan Africa** | Benin | 4,656(2,495-7,997) | 0.225(0.121-0.387) | 261(140-448) |
| **Sub-Saharan Africa** | Botswana | 3,687(2,327-5,876) | 0.300(0.189-0.478) | 1,246(786-1,985) |
| **Sub-Saharan Africa** | Burkina Faso | 7,429(4,255-12,164) | 0.315(0.180-0.515) | 236(135-386) |
| **Sub-Saharan Africa** | Burundi | 474(256-825) | 0.186(0.101-0.324) | 26(14-45) |

| | | | | |
|---|---|---|---|---|
| **Sub-Saharan Africa** | Cabo Verde | 475(256-827) | 0.378(0.203-0.657) | 758(407-1,318) |
| **Sub-Saharan Africa** | Cameroon | 10,148(5,191-18,457) | 0.242(0.124-0.441) | 267(137-485) |
| **Sub-Saharan Africa** | Central African Republic* | 448(250-774) | 0.249(0.139-0.430) | 68(38-118) |
| **Sub-Saharan Africa** | Chad* | 1,361(802-2,140) | 0.207(0.122-0.326) | 55(32-86) |
| **Sub-Saharan Africa** | Comoros | 317(149-573) | 0.342(0.160-0.617) | 272(127-491) |
| **Sub-Saharan Africa** | Congo, Dem. Rep. | 12,810(6,818-22,470) | 0.262(0.139-0.459) | 92(49-162) |
| **Sub-Saharan Africa** | Congo, Rep. | 905(462-1,611) | 0.260(0.133-0.462) | 114(58-202) |
| **Sub-Saharan Africa** | Côte d'Ivoire | 17,149(9,250-29,122) | 0.193(0.104-0.328) | 450(242-763) |
| **Sub-Saharan Africa** | Equatorial Guinea* | 685(384-1,171) | 0.217(0.122-0.372) | 325(182-555) |
| **Sub-Saharan Africa** | Eritrea* | 1,043(599-1,723) | 0.300(0.172-0.496) | 222(127-366) |
| **Sub-Saharan Africa** | Eswatini | 980(510-1,748) | 0.331(0.172-0.591) | 694(361-1,237) |
| **Sub-Saharan Africa** | Ethiopia | 46,943(27,412-77,960) | 0.219(0.128-0.364) | 293(171-487) |
| **Sub-Saharan Africa** | Gabon | 1,942(1,072-3,364) | 0.189(0.104-0.327) | 645(356-1,118) |
| **Sub-Saharan Africa** | Gambia, The | 800(380-1,445) | 0.319(0.152-0.577) | 222(105-401) |
| **Sub-Saharan Africa** | Ghana | 34,505(17,572-60,301) | 0.416(0.212-0.728) | 833(424-1,456) |
| **Sub-Saharan Africa** | Guinea | 8,611(4,182-15,456) | 0.383(0.186-0.687) | 446(217-801) |
| **Sub-Saharan Africa** | Guinea-Bissau | 496(275-851) | 0.300(0.166-0.514) | 181(100-311) |
| **Sub-Saharan Africa** | Kenya | 19,428(11,760-32,191) | 0.175(0.106-0.290) | 267(161-442) |
| **Sub-Saharan Africa** | Lesotho | 601(283-1,120) | 0.507(0.239-0.945) | 249(117-464) |
| **Sub-Saharan Africa** | Liberia* | 437(237-729) | 0.207(0.112-0.345) | 61(33-102) |
| **Sub-Saharan Africa** | Madagascar | 3,654(1,887-6,382) | 0.234(0.121-0.409) | 91(47-158) |
| **Sub-Saharan Africa** | Malawi* | 2,738(1,475-4,753) | 0.231(0.124-0.401) | 97(52-168) |
| **Sub-Saharan Africa** | Mali | 4,338(2,074-7,952) | 0.209(0.100-0.383) | 139(66-255) |
| **Sub-Saharan Africa** | Mauritania | 1,585(738-3,027) | 0.149(0.070-0.285) | 235(110-449) |
| **Sub-Saharan Africa** | Mauritius | 2,703(1,812-3,959) | 0.326(0.218-0.477) | 2,161(1,449-3,165) |
| **Sub-Saharan Africa** | Mozambique | 4,270(2,354-7,391) | 0.234(0.129-0.404) | 90(50-156) |
| **Sub-Saharan Africa** | Namibia | 1,460(739-2,757) | 0.253(0.128-0.478) | 448(227-847) |
| **Sub-Saharan Africa** | Niger | 2,828(1,551-4,803) | 0.159(0.087-0.270) | 66(36-113) |
| **Sub-Saharan Africa** | Nigeria | 32,591(20,060-52,037) | 0.108(0.067-0.173) | 109(67-175) |
| **Sub-Saharan Africa** | Rwanda | 6,517(3,322-11,654) | 0.361(0.184-0.645) | 363(185-649) |
| **Sub-Saharan Africa** | Sao Tome and Principe* | 103(57-176) | 0.314(0.172-0.533) | 344(188-585) |
| **Sub-Saharan Africa** | Senegal | 7,972(4,221-13,292) | 0.245(0.130-0.409) | 325(172-543) |
| **Sub-Saharan Africa** | Seychelles* | 698(429-1,095) | 0.699(0.430-1.098) | 6,769(4,166-10,627) |
| **Sub-Saharan Africa** | Sierra Leone | 558(297-971) | 0.152(0.081-0.264) | 53(28-93) |
| **Sub-Saharan Africa** | Somalia* | 1,620(906-2,713) | 0.228(0.127-0.382) | 66(37-110) |
| **Sub-Saharan Africa** | South Africa | 52,947(39,768-73,882) | 0.269(0.202-0.375) | 775(582-1,081) |
| **Sub-Saharan Africa** | South Sudan* | 1,711(993-2,858) | 0.239(0.139-0.399) | 111(64-186) |
| **Sub-Saharan Africa** | Sudan | 11,292(5,965-19,925) | 0.215(0.113-0.379) | 183(96-322) |

| | | | | |
|---|---|---|---|---|
| **Sub-Saharan Africa** | Tanzania | 33,373(16,974-60,647) | 0.363(0.185-0.659) | 363(185-660) |
| **Sub-Saharan Africa** | Togo | 3,013(1,587-5,203) | 0.332(0.175-0.573) | 258(136-445) |
| **Sub-Saharan Africa** | Uganda | 20,067(10,003-36,630) | 0.381(0.190-0.696) | 299(149-546) |
| **Sub-Saharan Africa** | Zambia | 7,636(3,921-13,647) | 0.348(0.179-0.622) | 272(140-486) |
| **Sub-Saharan Africa** | Zimbabwe | 4,995(2,666-8,802) | 0.340(0.181-0.599) | 259(138-457) |
| **Others** | Cook Islands* | 57(34-90) | 0.534(0.317-0.841) | 3,289(1,954-5,183) |
| **Others** | Niue* | 2(1-3) | 0.557(0.335-0.887) | 1,179(710-1,876) |
| **Others** | Palestine* | 2,960(1,897-4,318) | 0.319(0.204-0.466) | 425(272-620) |
| **Others** | Tokelau* | 1(1-2) | 0.415(0.234-0.694) | 777(438-1,300) |

Please note that results for countries marked with an asterisk in column 2 are imputed due to missing data.

**eTable 6. Total macroeconomic burden attributable to cancers in 2020–2050 using a discount rate of 0% for 204 countries, by World Bank region (in 2017 INT$)**

| Region | World Bank Country | Economic cost in millions of 2017 INT$ (lower and upper bound) | Percentage of total GDP in 2020–2050 (lower and upper bound) | Per capita loss in 2017 INT$ (lower and upper bound) |
|---|---|---|---|---|
| **East Asia & Pacific** | American Samoa* | 169(100-271) | 0.512(0.305-0.825) | 3,097(1,843-4,985) |
| **East Asia & Pacific** | Australia | 368,716(268,232-518,662) | 0.654(0.476-0.920) | 12,577(9,150-17,692) |
| **East Asia & Pacific** | Brunei Darussalam | 8,055(4,683-12,912) | 0.793(0.461-1.272) | 16,930(9,843-27,138) |
| **East Asia & Pacific** | Cambodia | 29,466(16,645-48,777) | 0.495(0.280-0.819) | 1,507(851-2,494) |
| **East Asia & Pacific** | China | 9,189,816(6,169,449-12,962,148) | 0.664(0.446-0.936) | 6,351(4,264-8,958) |
| **East Asia & Pacific** | Fiji | 1,480(704-2,689) | 0.325(0.155-0.591) | 1,491(709-2,709) |
| **East Asia & Pacific** | Guam* | 1,626(992-2,540) | 0.561(0.343-0.877) | 8,837(5,392-13,805) |
| **East Asia & Pacific** | Indonesia | 825,326(504,478-1,332,219) | 0.392(0.240-0.633) | 2,688(1,643-4,338) |
| **East Asia & Pacific** | Japan | 1,412,600(1,221,883-1,594,886) | 0.811(0.702-0.916) | 12,092(10,460-13,652) |
| **East Asia & Pacific** | Kiribati* | 73(42-121) | 0.608(0.350-1.008) | 493(284-817) |
| **East Asia & Pacific** | Korea, Dem. People's Rep.* | 11,348(6,602-18,528) | 0.567(0.330-0.926) | 427(248-697) |
| **East Asia & Pacific** | Korea, Rep. | 749,966(551,151-1,047,478) | 0.733(0.539-1.024) | 14,977(11,007-20,918) |
| **East Asia & Pacific** | Lao PDR | 16,836(9,109-28,194) | 0.413(0.224-0.692) | 1,972(1,067-3,302) |
| **East Asia & Pacific** | Malaysia | 272,750(128,795-496,536) | 0.518(0.245-0.944) | 7,341(3,467-13,365) |
| **East Asia & Pacific** | Marshall Islands* | 49(26-86) | 0.472(0.251-0.824) | 724(385-1,264) |
| **East Asia & Pacific** | Micronesia, Fed. Sts.* | 80(32-154) | 0.553(0.220-1.067) | 615(244-1,187) |
| **East Asia & Pacific** | Mongolia | 13,915(7,276-25,213) | 0.599(0.313-1.085) | 3,574(1,869-6,476) |
| **East Asia & Pacific** | Myanmar* | 64,359(44,518-94,892) | 0.420(0.291-0.620) | 1,083(749-1,597) |
| **East Asia & Pacific** | Nauru* | 23(14-38) | 0.403(0.233-0.656) | 2,142(1,235-3,487) |
| **East Asia & Pacific** | New Zealand | 86,626(63,949-118,615) | 0.808(0.596-1.106) | 16,412(12,116-22,472) |
| **East Asia & Pacific** | Northern Mariana Islands* | 506(308-807) | 0.814(0.496-1.299) | 8,244(5,020-13,157) |
| **East Asia & Pacific** | Palau* | 77(46-126) | 0.801(0.477-1.305) | 4,240(2,527-6,909) |
| **East Asia & Pacific** | Papua New Guinea* | 6,232(3,714-10,193) | 0.327(0.195-0.534) | 537(320-879) |
| **East Asia & Pacific** | Philippines | 308,797(184,026-475,714) | 0.449(0.267-0.691) | 2,395(1,427-3,690) |

| | | | | |
|---|---|---|---|---|
| **East Asia & Pacific** | Samoa* | 193(106-318) | 0.349(0.192-0.576) | 829(457-1,368) |
| **East Asia & Pacific** | Singapore | 97,583(75,890-127,181) | 0.374(0.291-0.488) | 15,514(12,065-20,220) |
| **East Asia & Pacific** | Solomon Islands* | 525(298-874) | 0.688(0.390-1.145) | 540(306-899) |
| **East Asia & Pacific** | Taiwan (Province of China)* | 434,627(227,833-811,039) | 0.760(0.399-1.419) | 18,412(9,652-34,357) |
| **East Asia & Pacific** | Thailand | 231,311(120,247-420,575) | 0.398(0.207-0.723) | 3,344(1,738-6,079) |
| **East Asia & Pacific** | Timor-Leste* | 658(350-1,030) | 0.358(0.190-0.560) | 391(208-611) |
| **East Asia & Pacific** | Tonga* | 152(88-251) | 0.543(0.316-0.895) | 1,262(734-2,081) |
| **East Asia & Pacific** | Tuvalu* | 19(11-31) | 0.485(0.276-0.819) | 1,339(762-2,260) |
| **East Asia & Pacific** | Vanuatu* | 162(88-269) | 0.389(0.212-0.646) | 380(207-630) |
| **East Asia & Pacific** | Vietnam | 511,329(269,347-899,423) | 0.687(0.362-1.209) | 4,857(2,558-8,543) |
| **Europe & Central Asia** | Albania | 13,380(6,780-23,697) | 0.716(0.363-1.269) | 4,966(2,516-8,795) |
| **Europe & Central Asia** | Andorra* | 1,394(860-2,148) | 0.838(0.517-1.291) | 17,964(11,081-27,690) |
| **Europe & Central Asia** | Armenia | 12,625(8,304-19,391) | 0.584(0.384-0.896) | 4,318(2,840-6,632) |
| **Europe & Central Asia** | Austria | 91,628(72,216-119,246) | 0.499(0.394-0.650) | 10,003(7,884-13,018) |
| **Europe & Central Asia** | Azerbaijan | 25,469(13,687-44,052) | 0.500(0.269-0.864) | 2,354(1,265-4,072) |
| **Europe & Central Asia** | Belarus | 28,651(14,367-57,594) | 0.512(0.257-1.029) | 3,153(1,581-6,339) |
| **Europe & Central Asia** | Belgium | 119,634(86,441-168,513) | 0.547(0.395-0.771) | 9,983(7,213-14,062) |
| **Europe & Central Asia** | Bosnia and Herzegovina | 19,093(11,323-30,715) | 0.824(0.489-1.325) | 6,337(3,758-10,194) |
| **Europe & Central Asia** | Bulgaria | 117,289(63,421-207,156) | 1.543(0.834-2.726) | 19,067(10,310-33,676) |
| **Europe & Central Asia** | Croatia | 29,533(16,658-53,314) | 0.551(0.311-0.994) | 7,880(4,444-14,225) |
| **Europe & Central Asia** | Cyprus | 7,930(5,571-11,352) | 0.383(0.269-0.548) | 6,126(4,303-8,769) |
| **Europe & Central Asia** | Czech Republic | 158,586(104,701-249,469) | 0.767(0.506-1.206) | 14,862(9,812-23,379) |
| **Europe & Central Asia** | Denmark | 91,309(65,874-129,833) | 0.648(0.468-0.922) | 15,074(10,875-21,433) |
| **Europe & Central Asia** | Estonia | 27,226(15,624-46,447) | 1.100(0.631-1.876) | 21,817(12,520-37,221) |
| **Europe & Central Asia** | Finland | 48,007(35,954-66,098) | 0.470(0.352-0.647) | 8,648(6,477-11,906) |
| **Europe & Central Asia** | France | 703,849(483,962-1,035,372) | 0.646(0.444-0.950) | 10,515(7,230-15,468) |
| **Europe & Central Asia** | Georgia | 10,463(6,666-15,454) | 0.335(0.214-0.495) | 2,777(1,770-4,102) |
| **Europe & Central Asia** | Germany | 1,811,781(1,329,802-2,493,186) | 1.105(0.811-1.520) | 21,985(16,136-30,253) |
| **Europe & Central Asia** | Greece | 42,109(31,539-56,286) | 0.402(0.301-0.538) | 4,333(3,245-5,792) |
| **Europe & Central Asia** | Greenland* | 1,016(586-1,694) | 0.842(0.486-1.404) | 18,145(10,464-30,251) |
| **Europe & Central Asia** | Hungary | 159,092(94,986-263,872) | 0.963(0.575-1.597) | 17,471(10,431-28,978) |
| **Europe & Central Asia** | Iceland | 6,418(4,416-9,037) | 0.648(0.446-0.913) | 17,640(12,136-24,838) |
| **Europe & Central Asia** | Ireland | 463,209(341,570-650,138) | 0.784(0.578-1.100) | 86,509(63,792-121,420) |
| **Europe & Central Asia** | Italy | 443,111(373,120-519,697) | 0.544(0.458-0.638) | 7,649(6,441-8,972) |
| **Europe & Central Asia** | Kazakhstan | 74,272(48,680-113,068) | 0.321(0.210-0.488) | 3,456(2,265-5,262) |

| | | | | |
|---|---|---|---|---|
| **Europe & Central Asia** | Kyrgyz Republic | 7,357(4,559-11,245) | 0.453(0.281-0.692) | 935(580-1,430) |
| **Europe & Central Asia** | Latvia | 22,462(11,438-43,321) | 0.818(0.416-1.577) | 13,530(6,890-26,094) |
| **Europe & Central Asia** | Lithuania | 43,089(27,400-68,085) | 0.819(0.521-1.294) | 18,004(11,449-28,448) |
| **Europe & Central Asia** | Luxembourg | 11,112(8,053-15,405) | 0.334(0.242-0.464) | 15,539(11,261-21,543) |
| **Europe & Central Asia** | Moldova | 11,215(7,176-16,705) | 0.653(0.418-0.973) | 2,995(1,916-4,460) |
| **Europe & Central Asia** | Monaco* | 5,492(3,556-8,449) | 1.432(0.927-2.202) | 128,325(83,083-197,409) |
| **Europe & Central Asia** | Montenegro | 6,749(4,075-10,667) | 1.176(0.710-1.859) | 10,979(6,628-17,352) |
| **Europe & Central Asia** | Netherlands | 339,278(249,059-460,841) | 0.852(0.626-1.158) | 19,535(14,340-26,534) |
| **Europe & Central Asia** | North Macedonia | 13,096(6,819-23,934) | 0.852(0.444-1.557) | 6,554(3,413-11,979) |
| **Europe & Central Asia** | Norway | 85,511(74,803-98,965) | 0.620(0.542-0.717) | 14,122(12,354-16,344) |
| **Europe & Central Asia** | Poland | 556,598(329,092-959,719) | 0.794(0.470-1.370) | 15,476(9,151-26,685) |
| **Europe & Central Asia** | Portugal | 60,021(41,461-85,936) | 0.432(0.298-0.618) | 6,182(4,270-8,851) |
| **Europe & Central Asia** | Romania* | 313,444(187,968-500,868) | 0.969(0.581-1.548) | 17,622(10,568-28,159) |
| **Europe & Central Asia** | Russian Federation | 659,930(430,858-990,590) | 0.532(0.347-0.798) | 4,675(3,053-7,018) |
| **Europe & Central Asia** | San Marino* | 721(396-1,264) | 0.926(0.509-1.624) | 21,072(11,582-36,950) |
| **Europe & Central Asia** | Serbia | 77,895(42,253-142,398) | 1.154(0.626-2.109) | 9,798(5,315-17,911) |
| **Europe & Central Asia** | Slovak Republic | 90,082(46,776-176,707) | 1.106(0.574-2.169) | 17,044(8,850-33,433) |
| **Europe & Central Asia** | Slovenia | 23,075(13,014-42,468) | 0.573(0.323-1.055) | 11,394(6,426-20,969) |
| **Europe & Central Asia** | Spain | 414,060(298,846-574,309) | 0.559(0.404-0.776) | 9,082(6,555-12,597) |
| **Europe & Central Asia** | Sweden | 131,077(107,879-162,843) | 0.556(0.458-0.691) | 12,139(9,990-15,080) |
| **Europe & Central Asia** | Switzerland | 159,103(114,033-223,727) | 0.670(0.480-0.942) | 17,051(12,221-23,977) |
| **Europe & Central Asia** | Tajikistan | 13,137(8,017-21,146) | 0.443(0.270-0.712) | 1,029(628-1,657) |
| **Europe & Central Asia** | Turkey | 499,577(299,685-816,137) | 0.346(0.207-0.565) | 5,464(3,278-8,926) |
| **Europe & Central Asia** | Turkmenistan* | 13,893(7,891-23,516) | 0.404(0.229-0.684) | 1,965(1,116-3,326) |
| **Europe & Central Asia** | Ukraine | 14,744(8,599-23,468) | 0.447(0.261-0.712) | 374(218-595) |
| **Europe & Central Asia** | United Kingdom | 755,204(661,571-860,416) | 0.658(0.577-0.750) | 10,584(9,271-12,058) |
| **Europe & Central Asia** | Uzbekistan | 126,298(74,924-194,097) | 0.664(0.394-1.021) | 3,254(1,931-5,001) |
| **Latin America & Caribbean** | Antigua and Barbuda* | 476(316-695) | 0.545(0.362-0.796) | 4,478(2,973-6,537) |
| **Latin America & Caribbean** | Argentina | 154,798(110,064-217,155) | 0.505(0.359-0.708) | 3,065(2,180-4,300) |
| **Latin America & Caribbean** | Bahamas, The | 3,160(1,872-5,037) | 0.710(0.421-1.131) | 7,252(4,297-11,559) |
| **Latin America & Caribbean** | Barbados | 1,097(657-1,730) | 0.840(0.503-1.324) | 3,831(2,294-6,040) |
| **Latin America & Caribbean** | Belize | 484(321-690) | 0.520(0.346-0.743) | 980(651-1,399) |
| **Latin America & Caribbean** | Bolivia | 25,101(13,347-41,979) | 0.529(0.281-0.885) | 1,807(961-3,022) |
| **Latin America & Caribbean** | Brazil | 276,052(233,914-329,771) | 0.284(0.241-0.339) | 1,227(1,040-1,466) |

| | | | | |
|---|---|---|---|---|
| **Latin America & Caribbean** | Chile | 92,127(66,430-127,809) | 0.490(0.353-0.680) | 4,653(3,355-6,456) |
| **Latin America & Caribbean** | Colombia | 137,538(70,615-251,995) | 0.420(0.215-0.769) | 2,542(1,305-4,658) |
| **Latin America & Caribbean** | Costa Rica | 29,847(16,102-50,950) | 0.575(0.310-0.982) | 5,388(2,907-9,197) |
| **Latin America & Caribbean** | Cuba* | 57,111(33,023-92,231) | 0.834(0.482-1.346) | 5,241(3,031-8,465) |
| **Latin America & Caribbean** | Dominica* | 228(141-361) | 0.805(0.498-1.274) | 3,145(1,945-4,978) |
| **Latin America & Caribbean** | Dominican Republic | 99,313(42,515-191,026) | 0.710(0.304-1.366) | 8,263(3,537-15,893) |
| **Latin America & Caribbean** | Ecuador | 20,385(12,436-33,312) | 0.313(0.191-0.512) | 985(601-1,609) |
| **Latin America & Caribbean** | El Salvador | 9,153(4,207-17,064) | 0.405(0.186-0.756) | 1,343(617-2,504) |
| **Latin America & Caribbean** | Grenada* | 535(372-755) | 0.678(0.472-0.958) | 4,626(3,219-6,535) |
| **Latin America & Caribbean** | Guatemala | 17,518(11,443-26,548) | 0.230(0.150-0.349) | 773(505-1,172) |
| **Latin America & Caribbean** | Guyana* | 10,587(5,873-18,036) | 0.455(0.252-0.774) | 12,881(7,146-21,945) |
| **Latin America & Caribbean** | Haiti* | 5,222(2,732-9,262) | 0.440(0.230-0.780) | 393(206-698) |
| **Latin America & Caribbean** | Honduras | 9,653(4,780-17,539) | 0.349(0.173-0.633) | 801(397-1,456) |
| **Latin America & Caribbean** | Jamaica | 6,202(3,141-10,740) | 0.668(0.338-1.157) | 2,052(1,039-3,553) |
| **Latin America & Caribbean** | Mexico | 267,951(184,773-370,906) | 0.296(0.204-0.409) | 1,855(1,279-2,568) |
| **Latin America & Caribbean** | Nicaragua* | 4,490(2,697-7,229) | 0.297(0.178-0.478) | 585(351-941) |
| **Latin America & Caribbean** | Panama | 25,184(14,572-42,798) | 0.386(0.224-0.656) | 4,883(2,825-8,298) |
| **Latin America & Caribbean** | Paraguay | 19,303(9,899-34,170) | 0.448(0.229-0.792) | 2,344(1,202-4,149) |
| **Latin America & Caribbean** | Peru | 47,892(26,689-87,353) | 0.267(0.149-0.487) | 1,290(719-2,353) |
| **Latin America & Caribbean** | Puerto Rico* | 15,451(8,625-27,290) | 0.532(0.297-0.940) | 5,619(3,137-9,925) |
| **Latin America & Caribbean** | St. Kitts and Nevis* | 335(200-520) | 0.631(0.377-0.979) | 6,003(3,584-9,311) |
| **Latin America & Caribbean** | St. Lucia* | 616(423-878) | 0.722(0.496-1.029) | 3,299(2,266-4,702) |
| **Latin America & Caribbean** | St. Vincent and the Grenadines* | 443(313-616) | 0.754(0.534-1.048) | 3,957(2,799-5,496) |
| **Latin America & Caribbean** | Suriname | 1,257(685-2,067) | 0.475(0.259-0.781) | 1,951(1,064-3,209) |
| **Latin America & Caribbean** | Trinidad and Tobago* | 4,670(2,373-8,783) | 0.494(0.251-0.930) | 3,349(1,702-6,299) |
| **Latin America & Caribbean** | Uruguay | 14,103(10,025-19,689) | 0.499(0.355-0.697) | 3,933(2,796-5,491) |
| **Latin America & Caribbean** | Venezuela, RB* | 71,498(34,608-133,895) | 0.531(0.257-0.995) | 2,095(1,014-3,923) |
| **Latin America & Caribbean** | Virgin Islands (U.S.)* | 1,741(1,098-2,746) | 0.899(0.567-1.418) | 17,960(11,331-28,331) |
| **Middle East & North Africa** | Algeria* | 48,166(28,513-78,232) | 0.253(0.150-0.410) | 911(539-1,480) |
| **Middle East & North Africa** | Bahrain | 4,789(3,357-7,094) | 0.148(0.104-0.220) | 2,303(1,615-3,412) |
| **Middle East & North Africa** | Djibouti | 1,585(743-3,103) | 0.343(0.161-0.672) | 1,366(641-2,675) |
| **Middle East & North Africa** | Egypt, Arab Rep. | 276,814(140,183-530,657) | 0.328(0.166-0.628) | 2,116(1,072-4,057) |
| **Middle East & North Africa** | Iran, Islamic Rep.* | 161,206(118,789-198,603) | 0.371(0.274-0.458) | 1,696(1,249-2,089) |
| **Middle East & North Africa** | Iraq | 48,557(31,909-74,334) | 0.242(0.159-0.370) | 876(575-1,341) |

| | | | | |
|---|---|---|---|---|
| **Middle East & North Africa** | Israel | 127,157(90,374-179,396) | 0.631(0.448-0.890) | 11,921(8,472-16,818) |
| **Middle East & North Africa** | Jordan | 7,749(4,544-12,934) | 0.177(0.104-0.296) | 682(400-1,138) |
| **Middle East & North Africa** | Kuwait | 13,821(10,789-18,358) | 0.208(0.162-0.276) | 2,810(2,193-3,732) |
| **Middle East & North Africa** | Lebanon | 1,366(738-2,368) | 0.260(0.141-0.451) | 213(115-370) |
| **Middle East & North Africa** | Libya* | 62,604(34,108-109,289) | 0.384(0.209-0.670) | 7,994(4,355-13,956) |
| **Middle East & North Africa** | Malta | 11,085(8,078-15,239) | 0.694(0.506-0.954) | 25,103(18,294-34,509) |
| **Middle East & North Africa** | Morocco | 27,802(16,944-46,282) | 0.221(0.135-0.368) | 659(402-1,097) |
| **Middle East & North Africa** | Oman | 9,652(4,815-19,669) | 0.149(0.074-0.303) | 1,572(784-3,204) |
| **Middle East & North Africa** | Qatar | 10,550(7,534-15,890) | 0.107(0.076-0.160) | 3,060(2,185-4,610) |
| **Middle East & North Africa** | Saudi Arabia | 142,904(96,671-219,452) | 0.219(0.148-0.336) | 3,522(2,383-5,409) |
| **Middle East & North Africa** | Syrian Arab Republic* | 7,913(4,619-13,057) | 0.315(0.184-0.519) | 287(168-474) |
| **Middle East & North Africa** | Tunisia | 11,520(7,347-17,930) | 0.253(0.162-0.394) | 887(566-1,380) |
| **Middle East & North Africa** | United Arab Emirates* | 122,914(57,200-229,699) | 0.410(0.191-0.766) | 11,702(5,446-21,868) |
| **Middle East & North Africa** | Yemen, Rep.* | 8,998(5,163-14,707) | 0.244(0.140-0.399) | 228(131-373) |
| **North America** | Bermuda* | 1,829(1,197-2,775) | 0.699(0.458-1.061) | 31,188(20,405-47,315) |
| **North America** | Canada | 538,023(388,202-757,460) | 0.759(0.548-1.069) | 12,801(9,236-18,021) |
| **North America** | United States | 7,784,167(6,886,149-9,028,381) | 0.906(0.802-1.051) | 21,787(19,273-25,269) |
| **South Asia** | Afghanistan* | 7,713(4,394-12,773) | 0.317(0.181-0.525) | 147(84-244) |
| **South Asia** | Bangladesh | 206,721(98,430-410,685) | 0.255(0.121-0.506) | 1,134(540-2,253) |
| **South Asia** | Bhutan | 1,809(795-3,487) | 0.333(0.146-0.641) | 2,111(927-4,070) |
| **South Asia** | India | 2,217,555(1,444,655-3,206,137) | 0.299(0.195-0.433) | 1,442(940-2,086) |
| **South Asia** | Maldives | 1,007(603-1,611) | 0.207(0.124-0.331) | 1,844(1,103-2,950) |
| **South Asia** | Nepal | 22,384(11,759-39,641) | 0.291(0.153-0.515) | 666(350-1,179) |
| **South Asia** | Pakistan | 321,654(187,921-524,010) | 0.513(0.300-0.836) | 1,142(667-1,860) |
| **South Asia** | Sri Lanka | 54,571(23,458-114,693) | 0.397(0.171-0.834) | 2,484(1,068-5,220) |
| **Sub-Saharan Africa** | Angola | 14,631(7,731-24,784) | 0.222(0.117-0.376) | 276(146-467) |
| **Sub-Saharan Africa** | Benin | 7,117(3,803-12,251) | 0.239(0.128-0.412) | 399(213-686) |
| **Sub-Saharan Africa** | Botswana | 5,576(3,528-8,883) | 0.325(0.206-0.518) | 1,883(1,192-3,000) |
| **Sub-Saharan Africa** | Burkina Faso | 11,600(6,638-19,016) | 0.338(0.194-0.555) | 368(211-604) |
| **Sub-Saharan Africa** | Burundi | 687(372-1,198) | 0.200(0.108-0.349) | 38(20-66) |
| **Sub-Saharan Africa** | Cabo Verde | 721(386-1,257) | 0.404(0.216-0.704) | 1,148(615-2,004) |
| **Sub-Saharan Africa** | Cameroon | 15,485(7,896-28,267) | 0.260(0.132-0.474) | 407(208-743) |
| **Sub-Saharan Africa** | Central African Republic* | 680(380-1,180) | 0.267(0.149-0.462) | 103(58-179) |
| **Sub-Saharan Africa** | Chad* | 1,959(1,151-3,081) | 0.222(0.130-0.349) | 79(46-124) |
| **Sub-Saharan Africa** | Comoros | 481(224-870) | 0.370(0.173-0.670) | 412(192-746) |
| **Sub-Saharan Africa** | Congo, Dem. Rep. | 19,520(10,387-34,236) | 0.278(0.148-0.487) | 140(75-246) |

| | | | | |
|---|---|---|---|---|
| **Sub-Saharan Africa** | Congo, Rep. | 1,219(620-2,175) | 0.276(0.140-0.493) | 153(78-273) |
| **Sub-Saharan Africa** | Côte d'Ivoire | 26,950(14,517-45,823) | 0.205(0.110-0.348) | 706(381-1,201) |
| **Sub-Saharan Africa** | Equatorial Guinea* | 891(498-1,526) | 0.233(0.130-0.399) | 422(236-723) |
| **Sub-Saharan Africa** | Eritrea* | 1,564(894-2,593) | 0.322(0.184-0.534) | 332(190-551) |
| **Sub-Saharan Africa** | Eswatini | 1,431(753-2,541) | 0.352(0.185-0.624) | 1,014(533-1,799) |
| **Sub-Saharan Africa** | Ethiopia | 74,687(43,466-124,249) | 0.231(0.134-0.383) | 466(271-776) |
| **Sub-Saharan Africa** | Gabon | 2,900(1,600-5,031) | 0.204(0.112-0.354) | 964(532-1,672) |
| **Sub-Saharan Africa** | Gambia, The | 1,235(585-2,234) | 0.343(0.162-0.620) | 343(162-620) |
| **Sub-Saharan Africa** | Ghana | 53,088(26,872-93,154) | 0.444(0.225-0.778) | 1,282(649-2,249) |
| **Sub-Saharan Africa** | Guinea | 13,527(6,538-24,343) | 0.406(0.196-0.731) | 701(339-1,262) |
| **Sub-Saharan Africa** | Guinea-Bissau | 755(418-1,296) | 0.318(0.176-0.546) | 276(153-473) |
| **Sub-Saharan Africa** | Kenya | 29,781(17,932-49,603) | 0.187(0.113-0.312) | 409(246-681) |
| **Sub-Saharan Africa** | Lesotho | 871(410-1,629) | 0.551(0.259-1.031) | 361(170-675) |
| **Sub-Saharan Africa** | Liberia* | 631(341-1,056) | 0.221(0.119-0.370) | 89(48-148) |
| **Sub-Saharan Africa** | Madagascar | 5,533(2,848-9,686) | 0.251(0.129-0.439) | 137(71-241) |
| **Sub-Saharan Africa** | Malawi* | 4,154(2,232-7,228) | 0.247(0.133-0.430) | 147(79-256) |
| **Sub-Saharan Africa** | Mali | 6,658(3,163-12,253) | 0.222(0.105-0.408) | 213(101-393) |
| **Sub-Saharan Africa** | Mauritania | 2,423(1,122-4,650) | 0.159(0.074-0.306) | 360(167-690) |
| **Sub-Saharan Africa** | Mauritius | 4,152(2,776-6,100) | 0.355(0.238-0.522) | 3,319(2,219-4,876) |
| **Sub-Saharan Africa** | Mozambique | 6,608(3,635-11,471) | 0.253(0.139-0.439) | 140(77-242) |
| **Sub-Saharan Africa** | Namibia | 2,146(1,079-4,084) | 0.275(0.138-0.524) | 659(331-1,254) |
| **Sub-Saharan Africa** | Niger | 4,437(2,425-7,549) | 0.171(0.093-0.291) | 104(57-177) |
| **Sub-Saharan Africa** | Nigeria | 46,414(28,537-74,290) | 0.113(0.069-0.181) | 156(96-249) |
| **Sub-Saharan Africa** | Rwanda | 10,215(5,190-18,304) | 0.381(0.193-0.682) | 569(289-1,019) |
| **Sub-Saharan Africa** | Sao Tome and Principe* | 156(85-266) | 0.336(0.183-0.575) | 518(282-886) |
| **Sub-Saharan Africa** | Senegal | 12,560(6,626-20,968) | 0.264(0.139-0.441) | 513(270-856) |
| **Sub-Saharan Africa** | Seychelles* | 1,071(655-1,693) | 0.753(0.461-1.190) | 10,395(6,359-16,431) |
| **Sub-Saharan Africa** | Sierra Leone | 810(430-1,413) | 0.164(0.087-0.286) | 77(41-135) |
| **Sub-Saharan Africa** | Somalia* | 2,452(1,368-4,111) | 0.244(0.136-0.410) | 99(55-167) |
| **Sub-Saharan Africa** | South Africa | 76,737(57,953-107,141) | 0.288(0.217-0.402) | 1,123(848-1,568) |
| **Sub-Saharan Africa** | South Sudan* | 2,568(1,487-4,294) | 0.257(0.149-0.429) | 167(97-279) |
| **Sub-Saharan Africa** | Sudan | 16,736(8,834-29,550) | 0.232(0.123-0.410) | 271(143-478) |
| **Sub-Saharan Africa** | Tanzania | 52,077(26,373-94,919) | 0.385(0.195-0.701) | 567(287-1,033) |
| **Sub-Saharan Africa** | Togo | 4,592(2,412-7,948) | 0.350(0.184-0.605) | 393(206-680) |
| **Sub-Saharan Africa** | Uganda | 31,064(15,375-57,016) | 0.406(0.201-0.745) | 463(229-849) |
| **Sub-Saharan Africa** | Zambia | 11,400(5,822-20,473) | 0.371(0.189-0.666) | 406(207-729) |
| **Sub-Saharan Africa** | Zimbabwe | 7,303(3,886-12,928) | 0.364(0.194-0.644) | 379(202-671) |
| **Others** | Cook Islands* | 86(51-136) | 0.573(0.338-0.909) | 4,929(2,908-7,822) |
| **Others** | Niue* | 3(2-5) | 0.598(0.358-0.959) | 1,769(1,058-2,835) |

| | | | | |
|---|---|---|---|---|
| **Others** | Palestine* | 4,446(2,836-6,509) | 0.343(0.219-0.502) | 638(407-934) |
| **Others** | Tokelau* | 2(1-3) | 0.445(0.249-0.749) | 1,165(653-1,961) |

Please note that results for countries marked with an asterisk in column 2 are imputed due to missing data.

### eTable 7. Total macroeconomic burden attributable to cancers in 2020–2050 using a discount rate of 3% for 204 countries, by World Bank region (in 2017 INT$)

| Region | World Bank Country | Economic cost in millions of 2017 INT$ (lower and upper bound) | Percentage of total GDP in 2020–2050 (lower and upper bound) | Per capita loss in 2017 INT$ (lower and upper bound) |
|---|---|---|---|---|
| **East Asia & Pacific** | American Samoa* | 92(55-147) | 0.459(0.276-0.731) | 1,695(1,019-2,701) |
| **East Asia & Pacific** | Australia | 203,325(148,778-283,598) | 0.582(0.426-0.812) | 6,936(5,075-9,674) |
| **East Asia & Pacific** | Brunei Darussalam | 4,500(2,643-7,164) | 0.688(0.404-1.095) | 9,459(5,554-15,057) |
| **East Asia & Pacific** | Cambodia | 15,032(8,520-24,835) | 0.450(0.255-0.743) | 769(436-1,270) |
| **East Asia & Pacific** | China | 4,983,836(3,381,179-6,974,828) | 0.598(0.406-0.838) | 3,444(2,337-4,820) |
| **East Asia & Pacific** | Fiji | 816(394-1,468) | 0.295(0.142-0.530) | 822(396-1,478) |
| **East Asia & Pacific** | Guam* | 890(549-1,377) | 0.503(0.310-0.777) | 4,840(2,983-7,484) |
| **East Asia & Pacific** | Indonesia | 431,819(265,647-692,304) | 0.351(0.216-0.563) | 1,406(865-2,255) |
| **East Asia & Pacific** | Japan | 806,176(699,214-907,865) | 0.719(0.624-0.810) | 6,901(5,985-7,771) |
| **East Asia & Pacific** | Kiribati* | 40(23-66) | 0.545(0.316-0.894) | 272(158-447) |
| **East Asia & Pacific** | Korea, Dem. People's Rep.* | 6,226(3,656-10,062) | 0.509(0.299-0.822) | 234(138-378) |
| **East Asia & Pacific** | Korea, Rep. | 410,919(303,081-569,958) | 0.653(0.481-0.905) | 8,206(6,053-11,382) |
| **East Asia & Pacific** | Lao PDR | 8,705(4,708-14,580) | 0.374(0.202-0.626) | 1,020(551-1,708) |
| **East Asia & Pacific** | Malaysia | 141,683(68,123-255,494) | 0.459(0.221-0.827) | 3,813(1,834-6,877) |
| **East Asia & Pacific** | Marshall Islands* | 27(14-47) | 0.423(0.227-0.731) | 397(213-686) |
| **East Asia & Pacific** | Micronesia, Fed. Sts.* | 45(18-86) | 0.495(0.199-0.944) | 347(139-662) |
| **East Asia & Pacific** | Mongolia | 7,509(3,957-13,490) | 0.548(0.289-0.985) | 1,929(1,016-3,465) |
| **East Asia & Pacific** | Myanmar* | 32,976(22,948-48,316) | 0.375(0.261-0.549) | 555(386-813) |
| **East Asia & Pacific** | Nauru* | 13(8-21) | 0.361(0.210-0.582) | 1,212(705-1,957) |
| **East Asia & Pacific** | New Zealand | 47,411(35,207-64,414) | 0.728(0.540-0.989) | 8,982(6,670-12,204) |
| **East Asia & Pacific** | Northern Mariana Islands* | 277(170-437) | 0.729(0.449-1.150) | 4,514(2,779-7,120) |
| **East Asia & Pacific** | Palau* | 44(27-71) | 0.719(0.434-1.158) | 2,428(1,463-3,908) |
| **East Asia & Pacific** | Papua New Guinea* | 3,347(2,011-5,438) | 0.292(0.176-0.475) | 289(173-469) |
| **East Asia & Pacific** | Philippines | 156,740(94,527-239,713) | 0.402(0.242-0.615) | 1,216(733-1,859) |
| **East Asia & Pacific** | Samoa* | 105(58-172) | 0.312(0.173-0.511) | 452(251-742) |
| **East Asia & Pacific** | Singapore | 53,095(41,390-68,936) | 0.334(0.260-0.433) | 8,441(6,580-10,960) |
| **East Asia & Pacific** | Solomon Islands* | 284(163-468) | 0.613(0.352-1.008) | 292(168-481) |
| **East Asia & Pacific** | Taiwan (Province of China)* | 237,914(126,042-438,462) | 0.680(0.360-1.254) | 10,079(5,339-18,574) |
| **East Asia & Pacific** | Thailand | 125,013(65,742-224,418) | 0.354(0.186-0.636) | 1,807(950-3,244) |
| **East Asia & Pacific** | Timor-Leste* | 376(202-585) | 0.320(0.171-0.498) | 223(120-347) |
| **East Asia & Pacific** | Tonga* | 84(50-138) | 0.485(0.284-0.792) | 701(411-1,145) |
| **East Asia & Pacific** | Tuvalu* | 9(5-16) | 0.434(0.249-0.726) | 683(393-1,143) |

| | | | | |
|---|---|---|---|---|
| **East Asia & Pacific** | Vanuatu* | 88(48-145) | 0.349(0.191-0.574) | 206(113-339) |
| **East Asia & Pacific** | Vietnam | 263,476(140,221-459,619) | 0.631(0.336-1.100) | 2,503(1,332-4,366) |
| **Europe & Central Asia** | Albania | 7,135(3,668-12,513) | 0.626(0.322-1.098) | 2,648(1,361-4,644) |
| **Europe & Central Asia** | Andorra* | 761(473-1,159) | 0.747(0.465-1.139) | 9,802(6,102-14,940) |
| **Europe & Central Asia** | Armenia | 6,849(4,541-10,420) | 0.537(0.356-0.817) | 2,343(1,553-3,564) |
| **Europe & Central Asia** | Austria | 51,494(40,583-66,859) | 0.447(0.352-0.580) | 5,621(4,430-7,299) |
| **Europe & Central Asia** | Azerbaijan | 14,871(8,091-25,428) | 0.453(0.246-0.774) | 1,374(748-2,350) |
| **Europe & Central Asia** | Belarus | 17,039(8,773-33,251) | 0.465(0.240-0.908) | 1,875(966-3,659) |
| **Europe & Central Asia** | Belgium | 67,713(49,211-94,563) | 0.491(0.357-0.686) | 5,650(4,106-7,891) |
| **Europe & Central Asia** | Bosnia and Herzegovina | 10,405(6,235-16,577) | 0.736(0.441-1.173) | 3,453(2,069-5,502) |
| **Europe & Central Asia** | Bulgaria | 62,490(34,372-108,718) | 1.350(0.742-2.348) | 10,159(5,588-17,674) |
| **Europe & Central Asia** | Croatia | 16,648(9,513-29,486) | 0.510(0.291-0.902) | 4,442(2,538-7,867) |
| **Europe & Central Asia** | Cyprus | 4,119(2,886-5,893) | 0.340(0.238-0.486) | 3,182(2,229-4,552) |
| **Europe & Central Asia** | Czech Republic | 85,285(56,567-132,827) | 0.685(0.454-1.067) | 7,992(5,301-12,448) |
| **Europe & Central Asia** | Denmark | 51,534(37,322-72,625) | 0.590(0.427-0.831) | 8,507(6,161-11,989) |
| **Europe & Central Asia** | Estonia | 14,521(8,431-24,463) | 0.974(0.566-1.642) | 11,636(6,756-19,603) |
| **Europe & Central Asia** | Finland | 26,908(20,187-36,842) | 0.418(0.313-0.572) | 4,847(3,636-6,637) |
| **Europe & Central Asia** | France | 396,669(275,220-576,502) | 0.576(0.400-0.837) | 5,926(4,112-8,613) |
| **Europe & Central Asia** | Georgia | 5,595(3,592-8,210) | 0.302(0.194-0.443) | 1,485(953-2,179) |
| **Europe & Central Asia** | Germany | 1,001,705(740,720-1,364,974) | 0.967(0.715-1.318) | 12,155(8,988-16,563) |
| **Europe & Central Asia** | Greece | 24,161(18,159-32,131) | 0.360(0.270-0.478) | 2,486(1,869-3,306) |
| **Europe & Central Asia** | Greenland* | 556(324-916) | 0.754(0.439-1.241) | 9,938(5,792-16,364) |
| **Europe & Central Asia** | Hungary | 86,952(52,679-142,084) | 0.879(0.533-1.436) | 9,549(5,785-15,603) |
| **Europe & Central Asia** | Iceland | 3,466(2,396-4,859) | 0.587(0.406-0.823) | 9,527(6,586-13,353) |
| **Europe & Central Asia** | Ireland | 229,062(168,987-320,431) | 0.723(0.533-1.011) | 42,780(31,560-59,844) |
| **Europe & Central Asia** | Italy | 251,184(211,981-293,712) | 0.482(0.407-0.563) | 4,336(3,659-5,070) |
| **Europe & Central Asia** | Kazakhstan | 41,871(27,722-63,009) | 0.295(0.196-0.445) | 1,948(1,290-2,932) |
| **Europe & Central Asia** | Kyrgyz Republic | 3,893(2,438-5,896) | 0.402(0.252-0.608) | 495(310-750) |
| **Europe & Central Asia** | Latvia | 12,246(6,353-23,133) | 0.732(0.379-1.382) | 7,377(3,827-13,934) |
| **Europe & Central Asia** | Lithuania | 23,354(14,964-36,512) | 0.731(0.469-1.143) | 9,758(6,253-15,256) |
| **Europe & Central Asia** | Luxembourg | 6,227(4,531-8,588) | 0.304(0.222-0.420) | 8,708(6,336-12,009) |
| **Europe & Central Asia** | Moldova | 6,103(3,949-9,003) | 0.589(0.381-0.868) | 1,630(1,054-2,404) |
| **Europe & Central Asia** | Monaco* | 2,991(1,960-4,536) | 1.275(0.835-1.933) | 69,886(45,790-105,993) |
| **Europe & Central Asia** | Montenegro | 3,595(2,193-5,633) | 1.046(0.638-1.638) | 5,849(3,567-9,164) |

| | | | | |
|---|---|---|---|---|
| **Europe & Central Asia** | Netherlands | 188,851(139,462-254,495) | 0.761(0.562-1.025) | 10,873(8,030-14,653) |
| **Europe & Central Asia** | North Macedonia | 7,235(3,845-12,961) | 0.769(0.409-1.377) | 3,621(1,924-6,487) |
| **Europe & Central Asia** | Norway | 48,746(42,641-56,302) | 0.558(0.488-0.645) | 8,050(7,042-9,298) |
| **Europe & Central Asia** | Poland | 298,210(179,459-503,967) | 0.716(0.431-1.210) | 8,292(4,990-14,013) |
| **Europe & Central Asia** | Portugal | 34,018(23,619-48,330) | 0.395(0.274-0.561) | 3,504(2,433-4,978) |
| **Europe & Central Asia** | Romania* | 165,381(100,287-260,851) | 0.865(0.525-1.364) | 9,298(5,638-14,665) |
| **Europe & Central Asia** | Russian Federation | 385,763(255,948-569,310) | 0.482(0.320-0.711) | 2,733(1,813-4,033) |
| **Europe & Central Asia** | San Marino* | 405(224-701) | 0.824(0.457-1.426) | 11,827(6,561-20,484) |
| **Europe & Central Asia** | Serbia | 42,219(23,283-75,768) | 1.030(0.568-1.849) | 5,310(2,929-9,530) |
| **Europe & Central Asia** | Slovak Republic | 47,724(25,182-91,769) | 0.963(0.508-1.852) | 9,029(4,764-17,363) |
| **Europe & Central Asia** | Slovenia | 12,835(7,308-23,206) | 0.527(0.300-0.953) | 6,338(3,609-11,459) |
| **Europe & Central Asia** | Spain | 229,061(165,837-315,770) | 0.502(0.364-0.693) | 5,024(3,637-6,926) |
| **Europe & Central Asia** | Sweden | 72,688(59,876-90,054) | 0.499(0.411-0.618) | 6,731(5,545-8,340) |
| **Europe & Central Asia** | Switzerland | 89,999(64,971-125,353) | 0.603(0.435-0.839) | 9,645(6,963-13,434) |
| **Europe & Central Asia** | Tajikistan | 6,600(4,029-10,608) | 0.394(0.240-0.633) | 517(316-831) |
| **Europe & Central Asia** | Turkey | 267,438(162,262-431,791) | 0.310(0.188-0.501) | 2,925(1,775-4,722) |
| **Europe & Central Asia** | Turkmenistan* | 7,967(4,554-13,390) | 0.362(0.207-0.608) | 1,127(644-1,894) |
| **Europe & Central Asia** | Ukraine | 9,803(5,870-15,348) | 0.356(0.213-0.558) | 248(149-389) |
| **Europe & Central Asia** | United Kingdom | 419,778(368,396-477,176) | 0.584(0.513-0.664) | 5,883(5,163-6,687) |
| **Europe & Central Asia** | Uzbekistan | 65,611(39,401-100,008) | 0.599(0.359-0.912) | 1,691(1,015-2,577) |
| **Latin America & Caribbean** | Antigua and Barbuda* | 250(168-362) | 0.486(0.326-0.703) | 2,355(1,578-3,409) |
| **Latin America & Caribbean** | Argentina | 88,425(63,411-122,880) | 0.442(0.317-0.614) | 1,751(1,256-2,433) |
| **Latin America & Caribbean** | Bahamas, The | 1,783(1,071-2,812) | 0.632(0.380-0.997) | 4,093(2,457-6,454) |
| **Latin America & Caribbean** | Barbados | 624(379-972) | 0.747(0.453-1.163) | 2,180(1,322-3,394) |
| **Latin America & Caribbean** | Belize | 267(179-380) | 0.458(0.306-0.650) | 542(362-770) |
| **Latin America & Caribbean** | Bolivia | 13,481(7,209-22,466) | 0.474(0.253-0.790) | 970(519-1,617) |
| **Latin America & Caribbean** | Brazil | 160,989(136,916-191,400) | 0.252(0.214-0.299) | 716(609-851) |
| **Latin America & Caribbean** | Chile | 50,429(36,585-69,484) | 0.429(0.311-0.591) | 2,547(1,848-3,510) |
| **Latin America & Caribbean** | Colombia | 74,992(39,317-135,039) | 0.371(0.194-0.668) | 1,386(727-2,496) |
| **Latin America & Caribbean** | Costa Rica | 15,915(8,715-26,879) | 0.506(0.277-0.854) | 2,873(1,573-4,852) |
| **Latin America & Caribbean** | Cuba* | 31,192(18,226-49,770) | 0.744(0.435-1.188) | 2,863(1,673-4,568) |
| **Latin America & Caribbean** | Dominica* | 129(81-202) | 0.717(0.447-1.123) | 1,782(1,111-2,791) |
| **Latin America & Caribbean** | Dominican Republic | 50,668(22,032-96,744) | 0.632(0.275-1.208) | 4,215(1,833-8,049) |
| **Latin America & Caribbean** | Ecuador | 11,470(7,046-18,565) | 0.274(0.168-0.444) | 554(340-897) |

| | | | | |
|---|---|---|---|---|
| **Latin America & Caribbean** | El Salvador | 4,987(2,354-9,155) | 0.356(0.168-0.654) | 732(346-1,344) |
| **Latin America & Caribbean** | Grenada* | 286(201-401) | 0.604(0.424-0.846) | 2,477(1,739-3,468) |
| **Latin America & Caribbean** | Guatemala | 9,389(6,164-14,133) | 0.204(0.134-0.307) | 415(272-624) |
| **Latin America & Caribbean** | Guyana* | 5,574(3,116-9,412) | 0.406(0.227-0.685) | 6,782(3,791-11,452) |
| **Latin America & Caribbean** | Haiti* | 2,960(1,560-5,207) | 0.391(0.206-0.689) | 223(118-392) |
| **Latin America & Caribbean** | Honduras | 5,125(2,541-9,291) | 0.308(0.153-0.559) | 426(211-771) |
| **Latin America & Caribbean** | Jamaica | 3,435(1,776-5,874) | 0.581(0.300-0.993) | 1,137(587-1,943) |
| **Latin America & Caribbean** | Mexico | 145,278(101,237-199,825) | 0.256(0.178-0.352) | 1,006(701-1,384) |
| **Latin America & Caribbean** | Nicaragua* | 2,532(1,529-4,060) | 0.265(0.160-0.426) | 330(199-529) |
| **Latin America & Caribbean** | Panama | 13,155(7,691-22,113) | 0.343(0.200-0.576) | 2,551(1,491-4,288) |
| **Latin America & Caribbean** | Paraguay | 10,331(5,383-18,083) | 0.392(0.204-0.686) | 1,254(654-2,196) |
| **Latin America & Caribbean** | Peru | 25,769(14,495-46,355) | 0.236(0.133-0.424) | 694(390-1,249) |
| **Latin America & Caribbean** | Puerto Rico* | 9,314(5,241-16,283) | 0.474(0.267-0.828) | 3,387(1,906-5,922) |
| **Latin America & Caribbean** | St. Kitts and Nevis* | 184(111-283) | 0.563(0.339-0.865) | 3,303(1,991-5,074) |
| **Latin America & Caribbean** | St. Lucia* | 343(238-485) | 0.643(0.446-0.908) | 1,837(1,273-2,594) |
| **Latin America & Caribbean** | St. Vincent and the Grenadines* | 244(174-336) | 0.671(0.479-0.924) | 2,180(1,554-3,000) |
| **Latin America & Caribbean** | Suriname | 718(397-1,172) | 0.415(0.229-0.677) | 1,114(616-1,819) |
| **Latin America & Caribbean** | Trinidad and Tobago* | 2,799(1,433-5,209) | 0.441(0.226-0.820) | 2,007(1,028-3,736) |
| **Latin America & Caribbean** | Uruguay | 8,056(5,763-11,166) | 0.447(0.320-0.619) | 2,247(1,607-3,114) |
| **Latin America & Caribbean** | Venezuela, RB* | 39,006(19,012-72,329) | 0.474(0.231-0.879) | 1,143(557-2,119) |
| **Latin America & Caribbean** | Virgin Islands (U.S.)* | 949(605-1,479) | 0.802(0.511-1.249) | 9,794(6,239-15,261) |
| **Middle East & North Africa** | Algeria* | 27,016(16,079-43,722) | 0.226(0.135-0.366) | 511(304-827) |
| **Middle East & North Africa** | Bahrain | 2,528(1,770-3,742) | 0.128(0.089-0.189) | 1,216(851-1,800) |
| **Middle East & North Africa** | Djibouti | 820(385-1,602) | 0.315(0.148-0.616) | 707(332-1,381) |
| **Middle East & North Africa** | Egypt, Arab Rep. | 143,860(73,679-272,886) | 0.290(0.149-0.551) | 1,100(563-2,086) |
| **Middle East & North Africa** | Iran, Islamic Rep.* | 91,367(67,921-112,229) | 0.332(0.247-0.408) | 961(714-1,180) |
| **Middle East & North Africa** | Iraq | 25,211(16,644-38,457) | 0.213(0.141-0.325) | 455(300-694) |
| **Middle East & North Africa** | Israel | 68,810(49,270-96,272) | 0.568(0.407-0.795) | 6,451(4,619-9,025) |
| **Middle East & North Africa** | Jordan | 4,299(2,546-7,095) | 0.158(0.093-0.260) | 378(224-624) |
| **Middle East & North Africa** | Kuwait | 7,637(5,955-10,140) | 0.178(0.138-0.236) | 1,552(1,211-2,061) |
| **Middle East & North Africa** | Lebanon | 884(486-1,511) | 0.213(0.117-0.365) | 138(76-236) |
| **Middle East & North Africa** | Libya* | 27,948(15,339-48,377) | 0.343(0.188-0.594) | 3,569(1,959-6,178) |
| **Middle East & North Africa** | Malta | 5,629(4,116-7,710) | 0.625(0.457-0.856) | 12,748(9,320-17,460) |
| **Middle East & North Africa** | Morocco | 15,086(9,180-25,064) | 0.197(0.120-0.327) | 358(218-594) |

| | | | | |
|---|---|---|---|---|
| **Middle East & North Africa** | Oman | 5,313(2,679-10,663) | 0.131(0.066-0.263) | 866(436-1,737) |
| **Middle East & North Africa** | Qatar | 5,762(4,126-8,632) | 0.092(0.066-0.139) | 1,672(1,197-2,504) |
| **Middle East & North Africa** | Saudi Arabia | 77,667(52,623-118,829) | 0.189(0.128-0.289) | 1,914(1,297-2,929) |
| **Middle East & North Africa** | Syrian Arab Republic* | 4,302(2,523-7,061) | 0.280(0.164-0.459) | 156(92-257) |
| **Middle East & North Africa** | Tunisia | 6,391(4,061-9,954) | 0.223(0.142-0.347) | 492(313-766) |
| **Middle East & North Africa** | United Arab Emirates* | 66,840(31,327-123,930) | 0.366(0.171-0.678) | 6,363(2,982-11,798) |
| **Middle East & North Africa** | Yemen, Rep.* | 4,924(2,840-8,025) | 0.219(0.126-0.356) | 125(72-204) |
| **North America** | Bermuda* | 997(658-1,497) | 0.623(0.412-0.936) | 17,004(11,225-25,528) |
| **North America** | Canada | 300,106(217,991-418,527) | 0.674(0.490-0.941) | 7,140(5,186-9,958) |
| **North America** | United States | 4,342,885(3,850,681-5,019,885) | 0.813(0.721-0.940) | 12,155(10,777-14,050) |
| **South Asia** | Afghanistan* | 4,375(2,504-7,210) | 0.282(0.162-0.465) | 84(48-138) |
| **South Asia** | Bangladesh | 106,149(50,945-209,462) | 0.235(0.113-0.464) | 582(279-1,149) |
| **South Asia** | Bhutan | 933(412-1,792) | 0.297(0.131-0.570) | 1,089(481-2,092) |
| **South Asia** | India | 1,141,492(750,688-1,639,549) | 0.273(0.179-0.392) | 743(488-1,066) |
| **South Asia** | Maldives | 508(304-813) | 0.183(0.110-0.293) | 930(557-1,488) |
| **South Asia** | Nepal | 11,611(6,113-20,499) | 0.261(0.137-0.460) | 345(182-610) |
| **South Asia** | Pakistan | 171,513(100,823-277,925) | 0.463(0.272-0.750) | 609(358-987) |
| **South Asia** | Sri Lanka | 29,655(13,078-61,101) | 0.357(0.157-0.736) | 1,350(595-2,781) |
| **Sub-Saharan Africa** | Angola | 8,658(4,603-14,619) | 0.200(0.106-0.337) | 163(87-275) |
| **Sub-Saharan Africa** | Benin | 3,785(2,032-6,493) | 0.218(0.117-0.373) | 212(114-364) |
| **Sub-Saharan Africa** | Botswana | 3,014(1,899-4,804) | 0.287(0.181-0.457) | 1,018(641-1,623) |
| **Sub-Saharan Africa** | Burkina Faso | 5,970(3,421-9,768) | 0.302(0.173-0.494) | 190(109-310) |
| **Sub-Saharan Africa** | Burundi | 396(214-690) | 0.179(0.097-0.311) | 22(12-38) |
| **Sub-Saharan Africa** | Cabo Verde | 388(209-674) | 0.364(0.196-0.632) | 619(333-1,074) |
| **Sub-Saharan Africa** | Cameroon | 8,257(4,231-14,988) | 0.233(0.119-0.423) | 217(111-394) |
| **Sub-Saharan Africa** | Central African Republic* | 365(205-630) | 0.239(0.134-0.413) | 55(31-96) |
| **Sub-Saharan Africa** | Chad* | 1,140(672-1,793) | 0.199(0.117-0.313) | 46(27-72) |
| **Sub-Saharan Africa** | Comoros | 259(122-468) | 0.327(0.153-0.589) | 222(104-401) |
| **Sub-Saharan Africa** | Congo, Dem. Rep. | 10,431(5,553-18,298) | 0.253(0.135-0.444) | 75(40-132) |
| **Sub-Saharan Africa** | Congo, Rep. | 786(401-1,396) | 0.252(0.129-0.447) | 99(50-175) |
| **Sub-Saharan Africa** | Côte d'Ivoire | 13,732(7,412-23,302) | 0.187(0.101-0.317) | 360(194-611) |
| **Sub-Saharan Africa** | Equatorial Guinea* | 602(338-1,029) | 0.209(0.117-0.357) | 286(160-488) |
| **Sub-Saharan Africa** | Eritrea* | 856(493-1,412) | 0.289(0.166-0.476) | 182(105-300) |
| **Sub-Saharan Africa** | Eswatini | 816(422-1,459) | 0.321(0.166-0.573) | 578(299-1,033) |
| **Sub-Saharan Africa** | Ethiopia | 37,327(21,838-61,930) | 0.213(0.124-0.353) | 233(136-387) |
| **Sub-Saharan Africa** | Gabon | 1,598(883-2,766) | 0.181(0.100-0.314) | 531(293-919) |
| **Sub-Saharan Africa** | Gambia, The | 647(308-1,167) | 0.307(0.146-0.554) | 179(85-324) |

| | | | | |
|---|---|---|---|---|
| **Sub-Saharan Africa** | Ghana | 27,949(14,280-48,735) | 0.402(0.205-0.701) | 675(345-1,177) |
| **Sub-Saharan Africa** | Guinea | 6,898(3,358-12,362) | 0.370(0.180-0.664) | 358(174-641) |
| **Sub-Saharan Africa** | Guinea-Bissau | 405(224-693) | 0.290(0.161-0.497) | 148(82-253) |
| **Sub-Saharan Africa** | Kenya | 15,769(9,573-26,054) | 0.169(0.103-0.279) | 216(131-358) |
| **Sub-Saharan Africa** | Lesotho | 502(237-935) | 0.484(0.229-0.903) | 208(98-388) |
| **Sub-Saharan Africa** | Liberia* | 365(198-609) | 0.199(0.108-0.332) | 51(28-86) |
| **Sub-Saharan Africa** | Madagascar | 2,986(1,545-5,209) | 0.226(0.117-0.394) | 74(38-129) |
| **Sub-Saharan Africa** | Malawi* | 2,234(1,205-3,875) | 0.222(0.120-0.385) | 79(43-137) |
| **Sub-Saharan Africa** | Mali | 3,520(1,689-6,437) | 0.202(0.097-0.369) | 113(54-206) |
| **Sub-Saharan Africa** | Mauritania | 1,288(602-2,453) | 0.144(0.067-0.274) | 191(89-364) |
| **Sub-Saharan Africa** | Mauritius | 2,192(1,472-3,205) | 0.310(0.208-0.454) | 1,752(1,177-2,562) |
| **Sub-Saharan Africa** | Mozambique | 3,447(1,903-5,957) | 0.224(0.123-0.386) | 73(40-126) |
| **Sub-Saharan Africa** | Namibia | 1,212(616-2,279) | 0.242(0.123-0.455) | 372(189-700) |
| **Sub-Saharan Africa** | Niger | 2,266(1,244-3,845) | 0.152(0.084-0.259) | 53(29-90) |
| **Sub-Saharan Africa** | Nigeria | 27,515(16,945-43,876) | 0.106(0.065-0.168) | 92(57-147) |
| **Sub-Saharan Africa** | Rwanda | 5,225(2,668-9,333) | 0.349(0.178-0.624) | 291(149-520) |
| **Sub-Saharan Africa** | Sao Tome and Principe* | 85(46-144) | 0.302(0.165-0.512) | 282(154-478) |
| **Sub-Saharan Africa** | Senegal | 6,373(3,381-10,618) | 0.235(0.125-0.392) | 260(138-433) |
| **Sub-Saharan Africa** | Seychelles* | 566(349-885) | 0.671(0.414-1.050) | 5,493(3,391-8,591) |
| **Sub-Saharan Africa** | Sierra Leone | 466(248-810) | 0.146(0.078-0.253) | 44(24-77) |
| **Sub-Saharan Africa** | Somalia* | 1,324(741-2,216) | 0.219(0.123-0.367) | 54(30-90) |
| **Sub-Saharan Africa** | South Africa | 44,282(33,166-61,771) | 0.259(0.194-0.362) | 648(485-904) |
| **Sub-Saharan Africa** | South Sudan* | 1,405(817-2,345) | 0.230(0.133-0.383) | 91(53-152) |
| **Sub-Saharan Africa** | Sudan | 9,332(4,932-16,460) | 0.206(0.109-0.363) | 151(80-266) |
| **Sub-Saharan Africa** | Tanzania | 26,824(13,676-48,664) | 0.351(0.179-0.636) | 292(149-530) |
| **Sub-Saharan Africa** | Togo | 2,453(1,294-4,231) | 0.322(0.170-0.556) | 210(111-362) |
| **Sub-Saharan Africa** | Uganda | 16,201(8,108-29,482) | 0.368(0.184-0.670) | 241(121-439) |
| **Sub-Saharan Africa** | Zambia | 6,288(3,238-11,206) | 0.336(0.173-0.599) | 224(115-399) |
| **Sub-Saharan Africa** | Zimbabwe | 4,159(2,224-7,310) | 0.327(0.175-0.575) | 216(115-379) |
| **Others** | Cook Islands* | 47(28-74) | 0.513(0.306-0.806) | 2,703(1,611-4,243) |
| **Others** | Niue* | 2(1-3) | 0.535(0.324-0.849) | 968(585-1,535) |
| **Others** | Palestine* | 2,429(1,560-3,536) | 0.306(0.197-0.446) | 349(224-507) |
| **Others** | Tokelau* | 1(1-2) | 0.399(0.226-0.665) | 638(361-1,064) |

Please note that results for countries marked with an asterisk in column 2 are imputed due to missing data.

**eFigure 6** illustrates the distribution of cancer types responsible for the most DALYs.

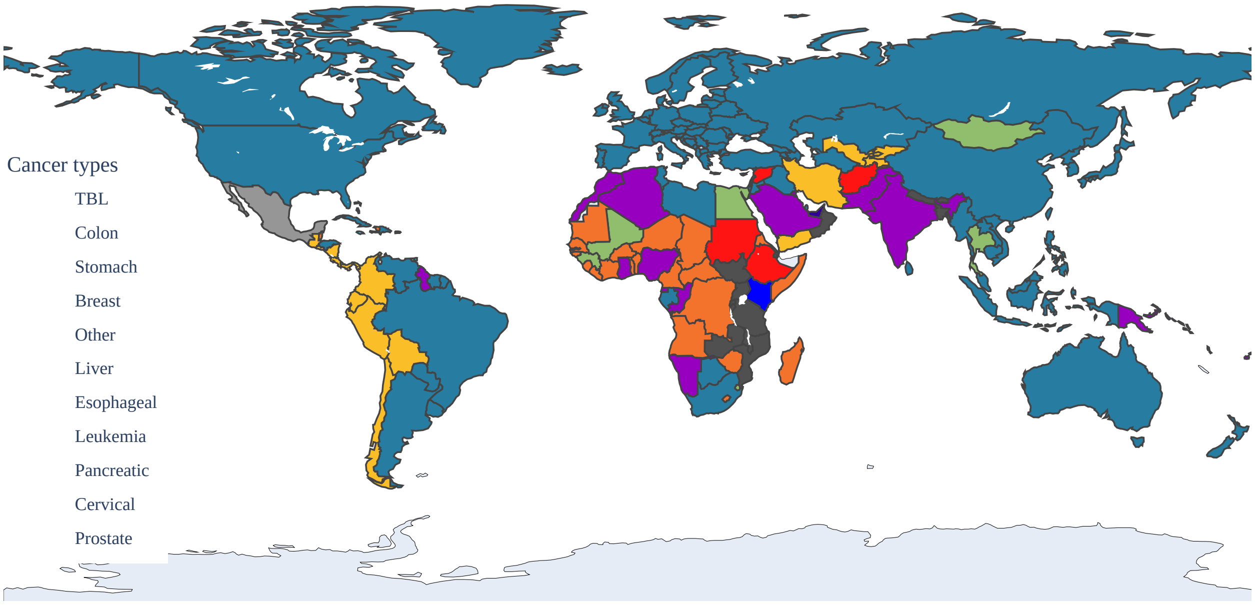


**eFigure 6. Cancer type responsible for the most DALYs in 2019 for each country (TBL = tracheal, bronchus, and lung)**

**eTables 8–9** illustrate the distribution of macroeconomic burden of cancers by World Bank region and income group with discount rates of 0% and 3%.

**eTable 8. Total macroeconomic cost, economic cost as a share of total GDP in 2020–2050, and per capita economic cost attributable to cancer mortality and morbidity, by World Bank region, by World Bank income group, and globally with discount rate of 0%**

| Region/income group | Economic cost in billions of 2017 INT$ (lower and upper bound) | Percentage of total GDP in 2020–2050 (lower and upper bound) | Per capita loss in 2017 INT$ (lower and upper bound) |
|---|---|---|---|
| **By World Bank region** | | | |
| **East Asia & Pacific** | 14,645(9,881-21,053) | 0.634(0.428-0.911) | 5,951(4,015-8,554) |
| **Europe & Central Asia** | 8,961(6,307-12,944) | 0.675(0.475-0.975) | 9,630(6,778-13,910) |
| **Latin America & Caribbean** | 1,432(931-2,184) | 0.380(0.247-0.580) | 1,994(1,297-3,041) |
| **Middle East & North Africa** | 1,107(672-1,806) | 0.312(0.189-0.508) | 1,971(1,197-3,215) |
| **North America** | 8,324(7,276-9,789) | 0.895(0.782-1.052) | 20,842(18,217-24,510) |
| **South Asia** | 2,833(1,772-4,313) | 0.311(0.195-0.474) | 1,342(840-2,043) |
| **Sub-Saharan Africa** | 600(342-1,011) | 0.251(0.143-0.423) | 366(208-616) |
| **By income group** | | | |
| **High income** | 18,964(15,113-24,582) | 0.770(0.614-0.998) | 15,307(12,199-19,842) |
| **Low income** | 259(141-444) | 0.278(0.152-0.477) | 269(147-462) |
| **Lower-middle income** | 5,629(3,397-8,925) | 0.348(0.210-0.551) | 1,430(863-2,267) |
| **Upper-middle income** | 12,979(8,496-19,013) | 0.574(0.376-0.841) | 4,898(3,206-7,175) |
| **Total** | 37,908(27,185-53,106) | 0.588(0.422-0.823) | 4,293(3,079-6,014) |

**eTable 9. Total macroeconomic cost, economic cost as a share of total GDP in 2020–2050, and per capita economic cost attributable to cancer mortality and morbidity, by World Bank region, by World Bank income group, and globally with discount rate of 3%**

| Region/income group | Economic cost in billions of 2017 INT$ (lower and upper bound) | Percentage of total GDP in 2020–2050 (lower and upper bound) | Per capita loss in 2017 INT$ (lower and upper bound) |
|---|---|---|---|
| **By World Bank region** | | | |
| **East Asia & Pacific** | 7,943(5,419-11,309) | 0.572(0.390-0.814) | 3,227(2,202-4,595) |
| **Europe & Central Asia** | 4,938(3,505-7,049) | 0.602(0.428-0.860) | 5,306(3,767-7,575) |
| **Latin America & Caribbean** | 791(522-1,191) | 0.333(0.220-0.501) | 1,102(727-1,658) |
| **Middle East & North Africa** | 592(364-956) | 0.275(0.169-0.443) | 1,054(647-1,702) |
| **North America** | 4,644(4,069-5,440) | 0.802(0.703-0.940) | 11,628(10,189-13,621) |
| **South Asia** | 1,466(925-2,218) | 0.284(0.179-0.430) | 695(438-1,051) |
| **Sub-Saharan Africa** | 323(185-540) | 0.227(0.130-0.379) | 196(113-329) |
| **By income group** | | | |
| **High income** | 10,524(8,430-13,531) | 0.688(0.551-0.884) | 8,494(6,805-10,922) |
| **Low income** | 135(74-232) | 0.252(0.139-0.432) | 141(77-241) |
| **Lower-middle income** | 2,935(1,787-4,621) | 0.315(0.192-0.495) | 745(454-1,174) |
| **Upper-middle income** | 7,064(4,679-10,245) | 0.514(0.341-0.746) | 2,666(1,766-3,866) |

## eAppendix 6. Differences in macroeconomic loss and lifetime disease burden

**eTable 10** illustrates the differences in economic costs across regions or income groups that also differ in terms of GDP, population, and DALYs.

**eTable 10. Comparison of macroeconomic loss and lifetime disease burden by World Bank region and country income group**

| Region/income group | Economic cost in billions of 2017 INT$ (global %) | DALYs in millions in 2020 (global %) | DALYs in millions in 2050 (global %) | Annual GDP in billions of 2017 INT$ in 2020–2050 (global %) | Annual POP in millions in 2020–2050 (global %) |
|---|---|---|---|---|---|
| **By World Bank region** | | | | | |
| **East Asia & Pacific** | 9,704(38.48%) | 99(39.13%) | 112(30.90%) | 52,763(35.70%) | 2,461(27.87%) |
| **Europe & Central Asia** | 5,993(23.76%) | 49(19.35%) | 49(13.57%) | 30,825(20.86%) | 930(10.54%) |
| **Latin America & Caribbean** | 960(3.81%) | 19(7.73%) | 31(8.42%) | 8,869(6.00%) | 718(8.13%) |
| **Middle East & North Africa** | 727(2.88%) | 9(3.43%) | 20(5.55%) | 8,163(5.52%) | 562(6.36%) |
| **North America** | 5,616(22.27%) | 19(7.43%) | 22(5.99%) | 21,720(14.70%) | 399(4.52%) |
| **South Asia** | 1,821(7.22%) | 40(15.91%) | 85(23.25%) | 19,983(13.52%) | 2,111(23.90%) |
| **Sub-Saharan Africa** | 395(1.57%) | 18(6.99%) | 45(12.22%) | 5,420(3.67%) | 1,642(18.59%) |
| **By World Bank income group** | | | | | |
| **High income** | 12,746(50.54%) | 63(25.13%) | 65(17.79%) | 57,446(38.87%) | 1,238(14.03%) |
| **Low income** | 168(0.66%) | 11(4.40%) | 28(7.77%) | 2,068(1.40%) | 962(10.90%) |
| **Lower-middle income** | 3,636(14.42%) | 73(29.05%) | 154(42.32%) | 35,950(24.32%) | 3,938(44.59%) |
| **Upper-middle income** | 8,619(34.18%) | 104(41.04%) | 115(31.54%) | 51,967(35.16%) | 2,650(30.01%) |
| **Sum** | 25,219(100.00%) | 252(100.00%) | 364(100.00%) | 147,771(99.98%) | 8,830(99.99%) |

Please note that each country is classified into World Bank regions as in **eTable 5.** The seven World Bank regions do not include Cook Islands, Niue, Palestine, and Tokelau.

## eAppendix 7. Contribution of treatment costs and human capital

**eFigure 7** shows the contribution of treatment costs to the total macroeconomic burden of cancers by country income group and by World Bank region. **eFigure 8** shows the contribution of human capital to the total macroeconomic burden of cancers by country income group and by World Bank region.

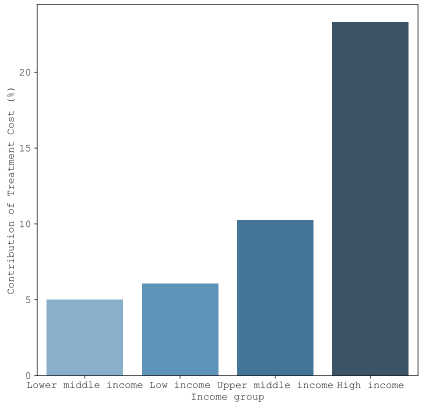


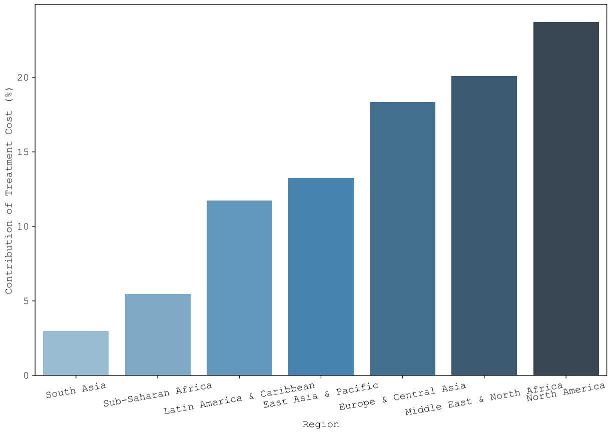


**eFigure 7. Contribution of treatment costs to the total economic cost of cancers by country income group and World Bank region**

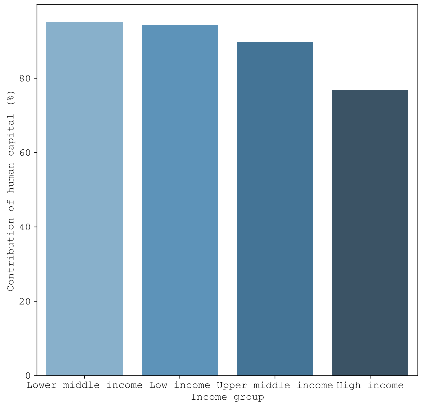


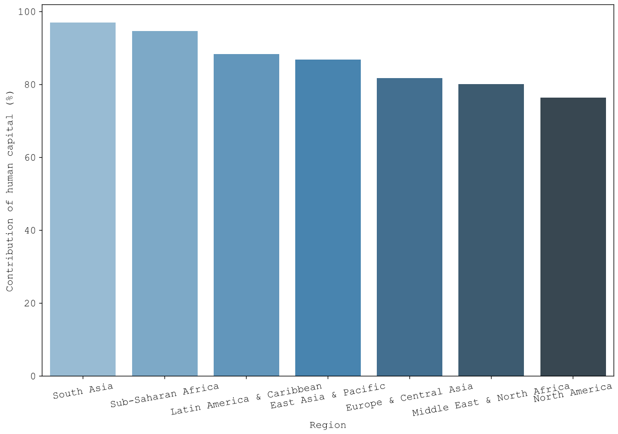


**eFigure 8. Contribution of human capital to the total economic cost of cancers by country income group and World Bank region**

## eAppendix 8. Strengths and limitations

Our model has several strengths and limitations, as summarized in **eTable 11**. First, we had to rely on imputations to calculate cancer-related health expenditures, which are based on data for the United States and then projected to other countries. This can either underestimate or overestimate the country-specific treatment costs for cancers. We also made assumptions about projections for labor participation rate, mortality, and morbidity data. This also may either underestimate or overestimate the results in some countries. Second, we had to impute the economic burden of cancers for 60 out of 204 countries and territories; however, this does not compromise our results, given that the 144 countries for which we had complete data account for 92.7% of the global population. Third, we did not account for changes in labor force participation of family members of people with cancer requiring informal care. With respect to this limitation, our findings should provide the lower bound of the economic cost of cancers. Finally, we did not consider unemployment, nor did we include an explicit treatment of price movements and endogenous savings in the framework.

Our analysis uses a simulation model grounded in dynamic macroeconomic theory to advance understanding of the global macroeconomic cost of cancers in several important ways. First, the calculations are based on recently developed methods and rely on the best available global data, including data from the recently updated Global Burden of Disease Study 2019, the Barro-Lee education database, the World Bank, and the International Labour Organization. Second, this study is the first to account for cancer's influence on

economic growth through a morbidity effect for 204 countries and territories in the world. Third, our framework is the first to consider productivity loss among people with different education and experience levels—the lack of which is a key limitation of previous studies. Fourth, our work shows the causal relationship between cancers and GDP. It avoids issues of reverse causality because we did not estimate the relationship but rather constructed it from our simulated production function. Fifth, we provided a detailed, step-by-step description of our methods and the data sources and specific parameters we used in our analysis in the SI Appendix. Sixth, we included sensitivity analyses to account for underlying uncertainty by adjusting the mortality and morbidity data based on upper and lower bounds of the GBD data.

**eTable 11. Strengths and limitations of our model**

| | Strengths | Limitations |
|---|---|---|
| **Data** | Rich global data from GBD, Barro-Lee education database, World Bank, and International Labour Organization | Robustness of the extrapolation of cancer-related health expenditures; assumptions on the projections for labor participation rate, mortality, and morbidity data |
| **Coverage** | 29 cancers and 204 countries | Missing data for 60 countries accounting for 7.3% of the total population |
| **Modeling** | Reproducible methods with mathematical description and sensitivity analyses that show the causal relationship between cancers and GDP; full consideration of morbidity effects and productivity loss among people with different education and experience levels; consideration of the effects of treatment costs on capital accumulation | Not accounting for reductions in the labor force participation of family members due to informal care for cancer; not accounting for unemployment; not accounting for behavioral responses; not including the explicit modeling of market price movements and endogenous savings |

## eReferences


1. GBD 2019 Diseases and Injuries Collaborators. Global burden of 369 diseases and injuries in 204 countries and territories, 1990-2019: a systematic analysis for the Global Burden of Disease Study 2019. *Lancet* 2020;396(10258):1204-22. doi: 10.1016/s0140-6736(20)30925-9 [published Online First: 2020/10/19]
2. Bloom DE, Chen S, Kuhn M, et al. The economic burden of chronic diseases: estimates and projections for China, Japan, and South Korea. *The Journal of the Economics of Ageing* 2020;17:100163.
3. Chen S, Kuhn M, Prettner K, et al. The macroeconomic burden of noncommunicable diseases in the United States: Estimates and projections. *PLOS ONE* 2018;13(11):e0206702. doi: 10.1371/journal.pone.0206702
4. Bloom DE, Chen S, Kuhn M, et al. The flip side of "live long and prosper": Noncommunicable diseases in the OECD and their macroeconomic impact. In: Bloom DE, ed. Live Long and Prosper? The Economics of Ageing Populations. London, UK: VoxEU.org and Centre for Economic Policy Research (CEPR), 2019:44.
5. Chen S, Kuhn M, Prettner K, et al. Noncommunicable Diseases Attributable To Tobacco Use In China: Macroeconomic Burden And Tobacco Control Policies. *Health Affairs* 2019;38(11):1832-39.
6. Chen S, Bloom DE. The macroeconomic burden of noncommunicable diseases associated with air pollution in China. *PLoS One* 2019;14(4):e0215663. doi: 10.1371/journal.pone.0215663 [published Online First: 2019/04/19]
7. Nargis N, Hussain AKMG, Asare S, et al. Economic loss attributable to cigarette smoking in the USA: an economic modelling study. *The Lancet Public Health* 2022;7(10):e834-e43. doi: 10.1016/S2468-2667(22)00202-X
8. Lucas RE. On the mechanics of economic development. *Journal of monetary economics* 1988;22(1):3-42.
9. Solow RM. A contribution to the theory of economic growth. *The Quarterly Journal of Economics* 1956;70(1):65–94.
10. Abegunde D, Stanciole A. An estimation of the economic impact of chronic noncommunicable diseases in selected countries Geneva, Switzerland: World Health Organization, Department of Chronic

Diseases and Health Promotion; 2006 [Available from: https://www.paho.org/bra/dmdocuments/WHO%20working_paper_Economic_impact_of_NCD.pdf accessed April 24 2022.
11. Mincer J. Schooling, experience, and earnings. Human Behavior & Social Institutions No. 2. 261 Madison Ave., New York, New York 10016: National Bureau of Economic Research Inc. 1974.
12. Chen S, Kuhn M, Prettner K, et al. The global macroeconomic burden of road injuries: estimates and projections for 166 countries. *The Lancet Planetary Health* 2019;3(9):e390-e98.
13. Barro RJ, Lee JW. A new data set of educational attainment in the world, 1950–2010. *Journal of Development Economics* 2013;104(September 2013):184–98. [published Online First: Feb 2016]
14. World Bank. World Bank database, GDP, PPP (constant 2017 international $) Washington, DC: World Bank; 2022 [Available from: https://data.worldbank.org/indicator/NY.GDP.MKTP.PP.KD accessed April 25 2022.
15. International Monetary Fund. World Economic Outlook Database 2022 [Available from: https://www.imf.org/external/pubs/ft/weo/2017/01/weodata/download.aspx accessed April 25 2022.
16. University of Groningen and University of California. Share of Labour Compensation in GDP at Current National Prices for United States [LABSHPUSA156NRUG], retrieved from FRED, Federal Reserve Bank of St. Louis Davis 2021 [Available from: https://fred.stlouisfed.org/series/LABSHPUSA156NRUG accessed April 25 2022.
17. International Labour Organization. Labour force by sex and age (thousands) Geneva, Switzerland: International Labour Organization; 2022 [Available from: http://ilo.org/global/statistics-and-databases/lang--en/index.htm accessed April 25 2022.
18. United Nations. 2019 Revision of World Population Prospects: Department of Economic and Social Affairs Population Dynamics; 2019 [Available from: https://population.un.org/wpp/Download/Standard/CSV/ accessed April 25 2022.
19. World Bank. World Bank database, gross savings (% of GDP) 2022 [Available from: https://data.worldbank.org/indicator/NY.GNS.ICTR.ZS accessed April 25 2022.
20. University of Groningen and University of California D. Capital stock at Current Purchasing Power Parities for United States [CKSPPPUSA666NRUG] retrieved from FRED, Federal Reserve Bank of St. Louis 2022 [Available from: https://fred.stlouisfed.org/series/CKSPPPUSA666NRUG accessed April 25 2022.
21. Grossmann V, Steger T, Trimborn T. Dynamically optimal R&D subsidization. *J Econ Dyn Control* 2013;37(3):516-34. doi: 10.1016/j.jedc.2012.10.007
22. Psacharopoulos G, Patrinos HA. Returns to investment in education: a decennial review of the global literature. *Education Economics* 2018;26(5):445-58. doi: https://doi.org/10.1080/09645292.2018.1484426
23. Heckman JJ, Lochner LJ, Todd PE. Earnings functions, rates of return and treatment effects: The Mincer equation and beyond. *Handbook of the Economics of Education* 2006;1(2006):307–458. doi: 10.1016/S1574-0692(06)01007-5
24. Guidry JJ, Aday LA, Zhang D, et al. Cost considerations as potential barriers to cancer treatment. *Cancer Practice* 1998;6(3):182-87.
25. Markman M, Luce R. Impact of the cost of cancer treatment: an internet-based survey. *Journal of Oncology Practice* 2010;6(2):69-73.
26. Smith TJ, Hillner BE. Bending the cost curve in cancer care. *The New England journal of medicine* 2011;364(21):2060.
27. Dieleman JL, Cao J, Chapin A, et al. US Health Care Spending by Payer and Health Condition, 1996-2016. *Jama* 2020;323(9):863-84. doi: 10.1001/jama.2020.0734 [published Online First: 2020/03/04]
28. Bloom DE, Cafiero E, Jané-Llopis E, et al. The global economic burden of noncommunicable diseases Geneva, World Economic Forum,2011 [Available from: https://www3.weforum.org/docs/WEF_Harvard_HE_GlobalEconomicBurdenNonCommunicableDiseases_2011.pdf accessed April 25 2022.
29. Ding D, Lawson KD, Kolbe-Alexander TL, et al. The economic burden of physical inactivity: a global analysis of major non-communicable diseases. *The Lancet* 2016;388(10051):1311-24.
