# Supplementary material for: Estimates and Projections of the Global Economic Cost of 29 Cancers in 204 Countries and Territories From 2020 to 2050
Source: JAMA Oncol. 2023 Feb 23;9(4):465–72. doi: 10.1001/jamaoncol.2022.7826 (PMC9951101; doi:10.1001/jamaoncol.2022.7826)
Supplement: Supplement 2. — Data Sharing Statement [file jamaoncol-e227826-s002.pdf]

Data Sharing Statement

Chen. Estimates and Projections of the Global Economic Cost of 29 Cancers in 204 Countries and Territories From 2020 to 2050. *JAMA Oncol*. Published February 23, 2023. doi:10.1001/jamaoncol.2022.7826

Data
Data available: Yes
Data types: Data (not involving human participants)
How to access data: simiao.chen@uni-heidelberg.de
When available: With publication

Supporting Documents
Document types: None

Additional Information
Who can access the data: Anyone requesting the data
Types of analyses: Any other than commercial purposes
Mechanisms of data availability: With investigator support
